# Study on biosynthesis pathway and accumulation mechanism of the dihydrochalcones in *Lithocarpus litseifolius*

**DOI:** 10.1093/hr/uhag061

**Published:** 2026-02-27

**Authors:** Yu-Si Yang, Yu-Ke Du, Jia-Li Li, Yong-Kang Wang, Cun-Yu Li, Xin-Qiang Zheng, Jian-Hui Ye, Yue-Rong Liang, Zhou-Tao Fang, Jian-Liang Lu

**Affiliations:** Tea Research Institute, Zhejiang University, Hangzhou 310058, China; Tea Research Institute, Zhejiang University, Hangzhou 310058, China; Tea Research Institute, Zhejiang University, Hangzhou 310058, China; Tea Research Institute, Zhejiang University, Hangzhou 310058, China; Tea Research Institute, Zhejiang University, Hangzhou 310058, China; Tea Research Institute, Zhejiang University, Hangzhou 310058, China; Tea Research Institute, Zhejiang University, Hangzhou 310058, China; Tea Research Institute, Zhejiang University, Hangzhou 310058, China; Tea Research Institute, Zhejiang University, Hangzhou 310058, China; Research and Development Department, Shaoxing Jianming Tea Industry Co., Ltd, Hangzhou 310058, China; Tea Research Institute, Zhejiang University, Hangzhou 310058, China

## Abstract

Dihydrochalcones (DHCs) are highly accumulated in tender leaves of *Lithocarpus litseifolius* but their biosynthetic pathway and accumulation mechanism remain unclear. In this study, candidate genes including one *cinnamoyl-CoA reductase* (*LlCCR*), two *double bond reductases* (*LlDBR1 ~ 2*), three *aldehyde hydrogenases* (*LlALDH1 ~ 3*), two *4-coumaroyl:CoA ligases* (*Ll4CL1 ~ 2*) and four *phloretin glycosyltransferases* (*LlP4′GT*, *LlP2′GT1 ~ 3*) were comprehensively investigated. The substrate specificities and catalytic kinetics of these gene-encoded enzymes were achieved. Through successive catalysis of LlALDH1, Ll4CL2, and chalcone synthase 1 (LlCHS1) or combined action of LlCCR and LlCHS1, phloretin was biosynthesized from direct precursor dihydro-*p*-coumaraldehyde, which had been converted from initial precursor *p*-coumaroyl-CoA by LlCCR-mediated carboxylic acid reduction and LlDBR1-catalyzed α,β-double bond saturation. High accumulation of the DHCs in tender leaves of *L. litseifolius* was mainly driven by efficient catalysis of LlCCR toward *p*-coumaroyl-CoA and highly expressed genes in the pathway, especially the *LlP4′GT* and *LlP2′GT1* which contributed to biosynthesis of trilobatin and phlorizin, respectively. Antisense oligodeoxyribonucleotide treatments against the *LlCCR*, *LlDBR1*, *LlALDH1*, *Ll4CL2*, *LlP4′GT,* and *LlP2′GT1* significantly reduced transcripts of the target genes and content of DHCs, confirming these genes might be involved in the pathway. This finding provides insight into the biosynthesis and accumulation mechanism of DHCs *in planta*.

## Introduction

Dihydrochalcones (DHCs), first identified from the bark of *Malus domestica*, are a type of non-classic flavonoid widely distributed in the *Malus* species, accounting for 2.8% to 10.1% of dry weight (DW) in leaf samples [1]. Phlorizin and trilobatin, common forms of DHCs, have been recognized as safe glycoside sweeteners contributing to a healthy, low-sugar diet [2]. In addition to the *Malus* species, DHCs were present at high levels in several medicinal plants, such as *Docynia dcne* [3], *Aspalathus linearis* [4], and *Lithocarpus litseifolius* [5]. Phlorizin has recently been detected in trace amounts across numerous plant species, suggesting the widespread presence of the DHC biosynthetic pathway in plants [6]. *L. litseifolius* was commonly known as sweet tea, as the tea prepared from its tender leaves or shoots has a sweet taste, conferred by DHCs [5, 7]. The DHC profile of *L. litseifolius* leaves is dominated by phlorizin and trilobatin, accounting for 21.08% to 32.52% of the DW [8]. The content of DHCs in *L. litseifolius* leaves is significantly higher than that in apples, making *L. litseifolius* an excellent natural source of DHCs and a wonderful model for elucidating the biosynthetic pathway.

DHCs are characterized by two C6 rings joined by a C3 bridge with a reduced α,β-double bond compared with chalcones. Various studies have attempted to clarify the biosynthetic pathway of DHC in *M. domestica*. The mainstream view holds that phloretin, the aglycone of DHCs, can be produced through a chalcone synthase (CHS)-catalyzed reaction in which three molecules of malonyl-CoA (MCoA) are cyclized with one molecule of dihydro-*p*-coumaroyl-CoA (dihydro-pCCoA) that is previously generated from *p*-coumaroyl-CoA (pCCoA) by catalysis of a double bond reductase (DBR) [9–11]. Phlorizin and trilobatin are then produced through glycosyl transferring reaction catalyzed by specific phloretin glycosyltransferases (PGTs) [12, 13]. However, conflicting results have been reported in the published researches regarding the formation of dihydro-pCCoA from pCCoA, a process that happens either directly or through some other intermediates. So far, three plant-derived enzymes from *M. domestica* have been reported to directly act on the α,β-double bond of pCCoA, namely hydroxycinnamoyl-CoA double bond reductase (MdHCDBR) and enoyl reductase-like 3 and 5 (MdENRL3/5) [10, 14]. The *MdHCDBR* was more recognized than *MdENRL3/5* as the functional gene in DHC biosynthesis. It has been reported that a MdHCDBR homolog in *Cannabis sativa*, CsDBR2, could catalyze the reduction of pCCoA with an activity 2-fold that of MdHCDBR [15]. Furthermore, Wang *et al*. [13] successfully synthesized trilobatin in *Nicotiana benthamiana* with MdHCDBR in their reconstituted pathway. However, replacing MdHCDBR with MdENRL3 caused a system failure. In contrast to the above results, some evidence challenged the role of MdENRL3/5 and MdHCDBR in phloretin synthesis. Caliandro *et al*. [16] detected no MdHCDBR enzyme activity with pCCoA. The downregulation of *MdHCDBR* or *MdENRL3* was not observed in chalcone isomerase (CHI)-overexpressed transgenic apple plants with reduced phlorizin biosynthesis [17]. Currently, the *de novo* biosynthesis of phloretin in a single strain was only accomplished by co-expressing *ScTSC13*, a *Saccharomyces cerevisiae* very long chain enoyl-CoA reductase capable of catalyzing pCCoA, along with genes involved in naringenin biosynthesis. However, the ratio of phloretin and naringenin remained unaffected when *ScTCS13* was replaced with *MdHCDBR* or *MdENRL3/5* [18]. The *de novo* biosynthesis approach was relatively low yielding and accompanied by naringenin generation and required endogenous microbial DBRs. Therefore, phloretin production in microorganisms was simplified by co-expressing *4-coumaroyl:CoA ligase* (*4CL*) and *CHS* while feeding dihydro-*p*-coumaric acid (dihydro-pCAci) to bypass the critical DBR step [19–21]. Based on the above results, host proteins like ScTSC13 might influence the heterologous production of phloretin in yeast or tobacco, and the catalytic activity of MdHCDBR and MdENRL3/5 toward pCCoA remained unclear. Very recently, an alternative pathway was described in apple with a branch point at naringenin chalcone by naringenin chalcone reductases (MdNCR1a/1b) to directly produce phloretin and the result was also supported *in planta*, as downregulation of *MdNCRs* in transgenic apple was accompanied by strongly reduced DHC levels in leaves [22].

Initially, DBR was thought to be responsible for detoxification of the lipid peroxide-derived reactive aldehydes by reducing the adjacent double bond [23, 24]. In addition to DHC, various studies have reported the involvement of DBR in the biosynthesis of many secondary metabolites, such as menthol [25], lignan [26], artemisinin [27], raspberry ketone [28], colchicine [29], bibenzyls [15], and perilla ketone [30]. What is more, DBRs from *Arabidopsis thaliana* [31], *N. benthamiana* [32] *M. domestica* [16], *Plagiochasma appendiculatum* [33], and *Marchantia paleacea* [34] also displayed catalytic activity for some specific hydroxycinnamaldehydes (HCAlds), namely *p*-coumaraldehyde (pCAld), coniferyl aldehyde (CAld), and sinapoyl aldehyde (SAld). A common feature of the DBR substrates mentioned above is that the double bond to be reduced needs to be activated by neighboring groups, such as ketones and nitro moieties [35, 36]. What is more, in colchicine synthesis, pCCoA was sequentially catalyzed by the cinnamoyl CoA reductase (CCR) and DBR to produce dihydro-*p*-coumaraldehyde (dihydro-pCAld), a molecule very similar to dihydro-pCCoA, with the coenzyme A (CoA) replaced with an aldehyde group [37]. Therefore, in our research, it was hypothesized that the critical double bond reduction step in DHC biosynthesis might occur at the level of HCAld instead of hydroxycinnamoyl-CoA (HCCoA), similar to colchicine synthesis.

To further elucidate the biosynthesis pathway and accumulation mechanism of DHCs in plants, several candidate genes including *CCRs*, *DBRs, ALDHs, 4CLs*, and *PGTs* were cloned from *L. litseifolius* and identified *in vitro* and *in vivo* in this study. The substrate specificities and catalytic kinetics of the prokaryotically expressed proteins encoded by these genes had been achieved. It was proved that phloretin could be biosynthesized through the successive catalysis of LlALDH1, Ll4CL2, and LlCHS1 or the action of LlCCR and LlCHS1 from the direct precursor dihydro-pCAld which was converted from the initial precursor pCCoA through carboxylic acid and α,β-double bond reductions catalyzed by LlCCR and LlDBR1, respectively, implying two pathways might co-exist during biosynthesis of phloretin and the α,β-double bond was saturated in pCAld instead of pCCoA. High accumulation of the DHCs in tender leaves of *L. litseifolius* was due to abundant metabolic flow mainly pulled by the efficient catalysis of LlCCR toward pCCoA and pushed by high expression of the relevant genes in the pathway, especially the *LlP4′GT* and *LlP2′GT1* which encoded the enzymes responsible for trilobatin and phlorizin production, respectively. These candidate genes involved in the DHC biosynthesis pathway were also verified by the treatment of antisense oligodeoxyribonucleotides (asODNs).

## Results

### The LlCCR pulls metabolic flow into DHCs

**Figure 1:**
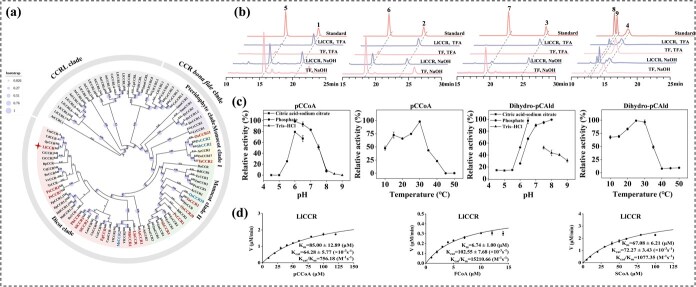
Biological characters of cinnamoyl CoA reductase (CCR) from *Lithocarpus litseifolius.* (a) Phylogenetic analysis of CCRs from *L. litseifolius* and other species according to the amino acid sequences. Information of all the CCRs was listed in Table S1. The bootstrap values were indicated by dots in different sizes, and the values on lines represented branch lengths. (b) Enzyme activity of recombinant LlCCR toward pCCoA, FCoA, sinapoyl-CoA (SCoA), and dihydro-*p*-coumaraldehyde (dihydro-pCAld). The expressed empty vector protein (TF) was used as control. The reactions were performed at 30°C and at pH 6.0 (forward reaction) or pH 8.0 (reverse reaction) for 30 min, and terminated by addition of trifluoroacetic acid (TFA) or NaOH. NaOH released the CoA esters as their acid forms in the reaction solution for HPLC detection. Signals were recorded at 280 nm on a 20 AD Shimadzu HPLC. (c) The effect of temperature and pH value on recombinant LlCCR activity toward pCCoA and dihydro-pCAld. Data were presented as mean ± SD of three repetitions. (d) Kinetic properties of recombinant LlCCR toward pCCoA, FCoA, and SCoA. The reactions were conducted under optimal conditions and terminated by addition of TFA. Data were presented as mean ± SD of three repetitions. Kinetic parameters were estimated after fitting the data to Michaelis–Menten equation. Peak 1, pCAld; peak 2, CAld; peak 3, SAld; peak 4, dihydro-pCAld; peak 5, p-coumaric acid; peak 6, ferulic acid; peak 7, sinapic acid; peak 8, dihydro-*p*-coumaryl alcohol; peak 9, dihydro-*p*-coumaric acid.

A total of 12 candidate *CCRs* (*LlCCR* and *LlCCRL1 ~ 11*) were cloned from *L. litseifolius* through RT-PCR. The phylogenetic analysis showed that all CCRs could be divided into two major clusters: CCR-like proteins (CCRLs) and *bona fide* CCRs (Fig. 1a). The *bona fide* CCRs were further classified into four groups: dicot clade, monocot clade I, monocot clade II, and *pteridophyte* clade. LlCCR was grouped within the dicot clade, closely related to CCRs from the *Fagaceae* family, while LlCCRL1 ~ 11 were grouped with *A. thaliana* and *bryophyte* CCRLs of unknown physiological functions. It should be noted that the names (*LlCCRL1 ~ 11*) were assigned only based on the order of gene identification and cloning in our experimental workflow because very few CCR-Like genes were functionally verified. In accordance with the phylogenetic analysis, only recombinant LlCCR displayed enzyme activity toward pCCoA, feruloyl-CoA (FCoA), and sinapoyl-CoA (SCoA) (Fig. S1, Fig. 1b), but the others (LlCCRL1 ~ 11) did not exhibit any activity toward these substrates (Fig. S2). In the temperature and pH tests, LlCCR showed the highest activity toward pCCoA at pH 6.0 in a 50 mM phosphate buffer and 30°C (Fig. 1c). The enzymatic kinetics of LlCCR for pCCoA, FCoA, and SCoA were determined at optimal pH value and temperature, and the result was shown in Fig. 1d. Based on the calculated *K*_cat_/*K*_m_ values, LlCCR exhibited the highest catalytic efficiency toward FCoA with a *K*_cat_/*K*_m_ of 15210.66 M^−1^⋅s^−1^, 14.11 and 20.12 times higher than that toward SCoA (1077.35 M^-1^⋅s^−1^) and pCCoA (756.18 M^-1^⋅s^−1^), in accordance with its conventional physiological function in lignin biosynthesis. The highest catalytic efficiency, as reflected by the *K*_cat_/*K*_m_ value of LlCCR toward FCoA, was mainly due to an extremely low *K*_m_ value (6.74 μM), much lower than those of pCCoA (85.00 μM) and SCoA (67.08 μM), indicating a high affinity toward FCoA.

A CCR from *Leucaena leucocephala* had been reported to catalyze a reverse reaction by converting the HCAld to HCCoA [38]. In our research, it was found that when dihydro-pCAld was incubated with LlCCR, NADP^+^ and CoA, a product peak of dihydro-pCAci was detected on the HPLC spectrum after being hydrolyzed with NaOH (Fig. 1b). Optimal acidity and temperature tests were performed, and relative enzyme activity was calculated by the peak area of dihydro-pCAci. LlCCR showed high activity toward dihydro-pCAld both at pH 8.0 in a 50 mM phosphate buffer and at pH 6.5 in a 50 mM citric acid-sodium citrate buffer, and LlCCR activity was high at 25°C, but greatly inhibited at temperatures above 35°C (Fig. 1c). Therefore, LlCCR could not only catalyze the reduction of HCCoA to corresponding aldehyde but also mediate the reverse oxidation process from dihydro-pCAld to dihydro-pCCoA. The kinetic properties of the LlCCR toward dihydro-pCAld was not determined due to the inevitable generation of dihydro-p-coumaroyl alcohol (dihydro-pCAhol) *in vitro*. It could be inferred that LlCCR not only played a role in lignin biosynthesis for its strong activity toward FCoA but also participated in DHC biosynthesis by catalyzing pCCoA.

### The LlDBRs act on saturation of α,β-double bond in HCAlds

**Figure 2:**
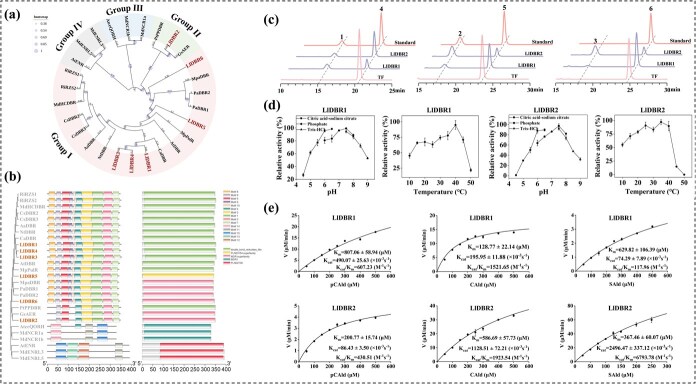
Biological characters of double bond reductase (DBR) from *Lithocarpus litseifolius.* (a) Phylogenetic analysis of DBRs from *L. litseifolius* and characterized DBRs from other species. Information of all the DBRs was listed in Table S2. The bootstrap values were indicated by dots in different sizes, and the values on lines represented branch lengths. (b) The conserved motifs identified by MEME Suite and the conserved domains identified by NCBI Batch CD-search in DBRs from *L. litseifolius* and other species. (c) Product detection in the reactions catalyzed by recombinant LlDBR1 and LlDBR2 toward *p*-coumaraldehyde (pCAld), coniferyl aldehyde (CAld) and sinapoyl aldehyde (SAld). The expressed empty vector protein (TF) was used as control. The reactions were performed at 30°C and pH 6.5 for 30 min and terminated by addition of trifluoroacetic acid (TFA). The product dihydro-*p*-coumaraldehyde (dihydro-pCAld), dihydro-coniferyl aldehyde (dihydro-CAld) and dihydro-sinapoyl aldehyde (dihydro-SAld) were recorded at 280 nm after HPLC separation. (d) The effect of temperature and pH value on recombinant LlDBR1 and LlDBR2 activity toward pCAld. Data were presented as mean ± SD of three repetitions. (e) Kinetic properties of recombinant LlDBR1 and LlDBR2 toward pCAld, CAld, and SAld. The reactions were performed under optimal conditions and terminated by addition of TFA. Data were presented as mean ± SD of three repetitions. Kinetic parameters were estimated after fitting the data to Michaelis–Menten equation. Peak 1, dihydro-pCAld; peak 2, dihydro-CAld; peak 3, dihydro-SAld; peak 4, pCAld; peak 5, CAld; peak 6, SAld.

Six *LlDBRs* (*LlDBR1 ~ 6*) had been separated and sequenced, the deduced amino acid sequences were subjected to the phylogenetic analysis. All the DBRs from *L. litseifolius* and other species were clustered into four groups (Fig. 2a). LlDBR1, LlDBR3, LlDBR4, LlDBR5, and LlDBR6 were in Group I, among them, the former three were close to CaDBR which was reported to reduce the α,β-double bond in pCAld during colchicine biosynthesis [29]. The LlDBR2 clustered with GsAER and PtPPDBR in Group II. GsAER from *Gloriosa superba* was used in heterologous colchicine production in tobacco [37], while PtPPDBR was involved in dihydrodehydrodiconiferyl alcohol biosynthesis and could catalyze CAld [26]. It was worth mentioning that all the *L. litseifolius* LlDBRs were distant from apple MdNCR1a/1b (Group III) which exhibited catalytic activity toward the double bond in naringenin chalcone [22]. Fifteen conserved motifs and five conserved domains were identified in all DBRs from *L. litseifolius* and other species (Fig. 2b). DBRs in Group I and II shared similar motif distribution with ten and seven motifs identified, respectively, while DBRs from Groups III and IV had completely different motifs. The enzyme activity of DBRs from *L. litseifolius* was investigated using pCAld, CAld, and SAld as the substrates, only recombinant LlDBR1 and LlDBR2 exhibited catalysis capability (Fig. S1, Fig. 2c), but LlDBR3 ~ 6 did not (Fig. S3). LlDBR1 and LlDBR2 showed high enzyme activities toward pCAld both at pH 6.5 in a 50 mM citric acid-sodium citrate buffer and pH 7.5 in a 50 mM phosphate buffer, and at 40°C and 35°C, respectively (Fig. 2d). The enzymatic kinetics of LlDBR1 and LlDBR2 were determined under optimal conditions, and distinct catalytic properties for substrates pCAld, CAld, and SAld were observed (Fig. 2e). LlDBR1 exhibited higher catalytic efficiency toward CAld (1521.65 M^−1^⋅s^−1^) and pCAld (607.23 M^−1^⋅s^−1^) than SAld (117.96 M^−1^⋅s^−1^). In contrast, LlDBR2 showed the highest catalytic efficiency for SAld (6793.78 M^−1^⋅s^−1^), followed by CAld (1923.54 M^−1^⋅s^−1^) and pCAld (430.51 M^−1^⋅s^−1^), indicating a strong and specific activity toward SAld. LlDBR1 and LlDBR2 showed similar catalytic efficiency for pCAld and CAld as reflected by the *K*_cat_/*K*_m_ values. However, the *K*_cat_/*K*_m_ value of LlDBR2 for SAld was 58 times higher than that of LlDBR1. In terms of *K*_cat_ values, LlDBR1 exhibited the highest conversion rate for pCAld (490.07 × 10^−3^ s^−1^), 2.50 and 6.60 times that of CAld (195.95 × 10^−3^ s^−1^) and SAld (74.29 × 10^−3^ s^−1^), respectively. On the contrary, LlDBR2 showed the highest value for SAld (2496.47 × 10^−3^ s^−1^), 2.21 and 28.89 times that of CAld (1128.51 × 10^−3^ s^−1^) and pCAld (86.43 × 10^−3^ s^−1^), respectively. Despite the highest *K*_cat_ values for pCAld, the reduction efficiency of LlDBR1 toward pCAld was lower than CAld as LlDBR1 possessed the highest *K*_m_ value for pCAld (807.06 μM) and the lowest one for CAld (128.77 μM). The *K*_cat_ value of LlDBR1 for pCAld was 5.7 times that of LlDBR2; however, the *K*_m_ value of LlDBR2 for pCAld (200.77 μM) was quite low, resulting in a *K*_cat_/*K*_m_ value close to LlDBR1. In summary, LlDBR1 preferred to catalyze the reduction of CAld and pCAld instead of SAld, and its highest *K*_cat_ was observed for pCAld; LlDBR2 showed high activity toward SAld and CAld instead of pCAld. Thus, the LlDBR1 might be involved in DHC biosynthesis *in vivo*.

### The LlALDHs play roles in the dihydro-pCAci generation

**Figure 3:**
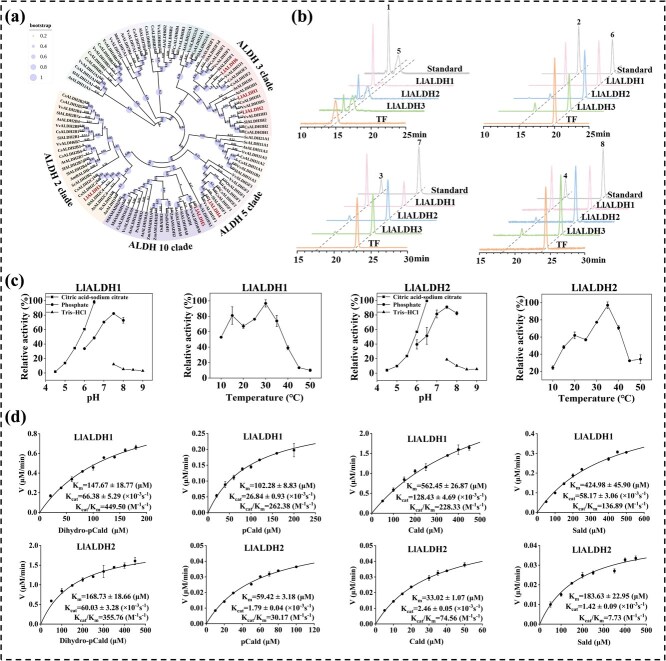
Biological characters of aldehyde dehydrogenases (ALDHs) from *Lithocarpus litseifolius.* (a) Phylogenetic analysis of ALDHs from *L. litseifolius* and other species. Information of all the ALDHs was listed in Table S3. The bootstrap values were indicated by dots in different sizes, and the values on lines represented branch lengths. (b) Enzyme activity of the recombinant LlALDH1-3 toward dihydro-*p*-coumaraldehyde (dihydro-pCAld), *p*-coumaraldehyde (pCAld), coniferaldehyde (CAld) and sinapaldehyde (SAld). The expressed empty vector protein (TF) was used as control. The reactions were performed at 30°C and pH 6.5 for 30 min, and terminated by addition of trifluoroacetic acid (TFA). The product dihydro-*p*-coumaric acid (dihydro-pCAci), *p*-coumaric acid (pCAci), ferulic acid (FAci), and sinapic acid (SAci) were recorded at 280 nm on a 20 AD Shimadzu HPLC. (c) The effect of temperature and pH on recombinant LlALDH1–2 activity toward dihydro-pCAld. Data were presented as mean ± SD of three repetitions. (d) Kinetic properties of recombinant LlALDH1 and LlALDH2 toward dihydro-pCAld, pCAld, CAld and SAld. The reactions were performed under optimal conditions and terminated by addition of TFA. Data were presented as mean ± SD of three repetitions. Kinetic parameters were estimated after fitting the data to Michaelis–Menten equation. Peak 1, dihydro-pCAci; peak 2, pCAci; peak 3, FAci; peak 4, SAci; peak 5, dihydro-pCAld; peak 6, pCAld; peak 7, CAld; peak 8, SAld.

Six *LlALDHs* (*LlALDH1 ~ 6*) with relatively high expression levels were screened out from the transcriptome of *L. litseifolius* by TBtools and cloned through RT-PCR. Phylogenetic tree was constructed according to the amino acid sequences and shown in Fig. 3a. In ALDH3 clade, *L. litseifolius* LlALDH1 and LlALDH2 clustered with AtALDH3H1 and AtALDH3I1 while LlALDH6 was close to AtALDH3F1. LlALDH3 was near to AtALDH2C4 in the ALDH2 clade, while LlALDH4 and LlALDH5 were grouped within the ALDH5 clade and the ALDH10 clade, respectively. The catalytic activities of the recombinant LlALDH1 ~ 6 were analyzed using dihydro-pCAld, pCAld, CAld, and SAld as substrates, the recombinant LlALDH4 ~ 6 did not exhibit activities toward these substrates (Fig. S4), while LlALDH1, LlALDH2, and LlALDH3 displayed enzyme activities, with the former two possessing relatively high activity toward dihydro-pCAld (Fig. S1, Fig. 3b). The optimal acidity and temperature were determined using dihydro-pCAld as the substrate, and LlALDH1 and LlALDH2 showed the highest catalytic activity both at pH 6.5 in a 50 mM phosphate buffer while at 30°C and 35°C for optimal temperature, respectively (Fig. 3c). Enzyme kinetic analysis was conducted for LlALDH1 and LlALDH2 and the result was shown in Fig. 3d. According to the calculated *K*_cat_/*K*_m_ values, they both displayed the highest catalytic activities toward dihydro-pCAld. The catalytic efficiency of LlALDH1 for dihydro-pCAld (449.50 M^−1^⋅s^−1^) was 1.71, 1.97, and 3.28 times that for pCAld (262.38 M^−1^ ⋅s^−1^), CAld (228.33 M^−1^ ⋅s^−1^), and SAld (136.89 M^−1^⋅s^−1^), respectively, and the catalytic efficiency of LlALDH2 for dihydro-pCAld (355.76 M^−1^⋅s^−1^) was 5.99, 10.77, and 46.02 times that for pCAld (59.42 M^−1^⋅s^−1^), CAld (33.02 M^−1^⋅s^−1^), and SAld (7.73 M^−1^⋅s^−1^). Comparatively, both LlALDH1 and LlALDH2 preferred dihydro-pCAld, but the catalytic efficiency of LlALDH1 was higher than that of LlALDH2.

### Two Ll4CLs catalyze the formation of HCCoAs

**Figure 4:**
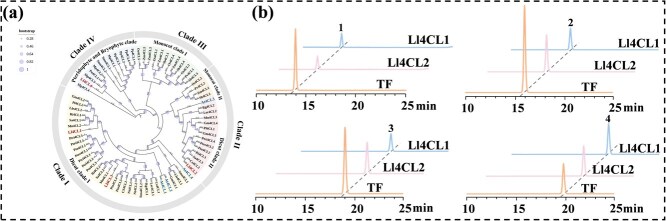
Biological characters of 4-coumaroyl:CoA ligases (4CLs) from *Lithocarpus litseifolius.* (a) Phylogenetic analysis of 4CLs from *L. litseifolius* and other species. Information of all the 4CLs was listed in Table S4. The bootstrap values were indicated by dots in different sizes, and the values on lines represented branch lengths. (b) Enzyme activity of Ll4CL1 and Ll4CL2 toward dihydro-*p*-coumaric acid (dihydro-pCAci), *p*-coumaric acid (pCAci), ferulic acid (FAci), and sinapic acid (SAci). The expressed empty vector protein (TF) was used as control. The reactions were performed at 30°C and pH 7.5 for 30 min and terminated by addition of trifluoroacetic acid. Enzyme activity was detected by monitoring the decrease of the substrates at 280 nm on a 20 AD Shimadzu HPLC. Peak 1, dihydro-pCAci; peak 2, pCAci; peak 3, FAci; peak 4, SAci.

Four candidate *4CL*s (*Ll4CL1 ~ 4*) were screened out from the transcriptome of *L. litseifolius* by TBtools and confirmed through RT-PCR. The phylogenetic tree was constructed according to the amino acid sequences, and all 4CLs were divided into four major clades (Fig. 4a). *L. litseifolius* Ll4CL1 and Ll4CL3 were clustered in dicot clade I together with At4CL1. Clade II was divided into the dicot and monocot subclades, and Ll4CL2 was clustered in dicot subclade with At4CL3. Ll4CL4 was included in Clade IV with many 4CLs from *pteridophyte* and *bryophyte.* Notably, although four to five members of 4CL had been identified in most monocot and dicot plants, only one 4CL from each species was clustered in Clade II, except for soybean Gm4CL3 and Gm4CL4, suggesting that 4CLs from Clade II might have different structures and functions in comparison with those from Clade I and Clade III. It had been reported that 4CLs mainly played two roles, one was involved in lignin biosynthesis or in the formation of cell-wall-bound phenylpropanoid derivatives, such as At4CL1 and Ptre4CL1 in dicot clade I, while another was responsible for flavonoid biosynthesis, such as At4CL3 and Ptre4CL2 in dicot clade II [39–41]. Therefore, the Ll4CL1 and Ll4CL3 might be associated with former, while Ll4CL2 likely belong to latter. Ll4CL1 and Ll4CL2 were selected for enzyme activity assays, as Ll4CL3 and Ll4CL4 showed extremely low transcript levels across all tested tissues of *L. litseifolius* (Fig. S5). Four candidate substrates, including dihydro-pCAci, *p*-coumaric acid (pCAci), ferulic acid (FAci), and sinapic acid (SAci), were tested. According to the decrease in substrates, both recombinant Ll4CL1 and Ll4CL2 exhibited high efficiency toward dihydro-pCAci and also showed catalytic activity for pCAci and FAci, but no activity for SAci (Fig. S1 and Fig. 4b).

### Phloretin can be generated through two ways from dihydro-pCAld

**Figure 5:**
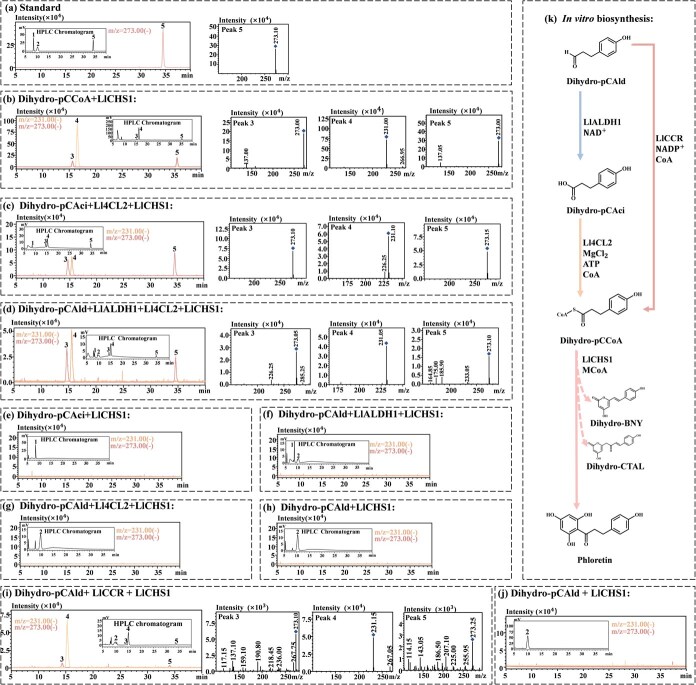
*In vitro* biosynthesis of phloretin from dihydro-*p*-coumaraldehyde (dihydro-pCAld) or dihydro-*p*-cinnamic acid (dihydro-pCAci) catalyzed by LlALDH1, Ll4CL2 and LlCHS1, or LlCCR and LlCHS1. (a) Dihydro-pCAld, dihydro-pCAci and phloretin standards on HPLC chromatogram, as well as the extracted ion chromatogram (EIC) and MS signal spectrum of phloretin. (b–h) HPLC chromatograms, EICs, and MS spectra of the catalytic products in different reaction systems; (b) Reaction system 1: including the substrates dihydro-*p*-coumaroyl-CoA (dihydro-pCCoA) and malonyl-CoA (MCoA), and the enzyme LlCHS1; (c) Whole reaction system 2: including the substrates dihydro-pCAci and MCoA, cofactors MgCl_2_, ATP and CoA, as well as the enzymes Ll4CL2 and LlCHS1; (d) Whole reaction system 3: including the substrates dihydro-pCAld and MCoA, cofactors NAD^+^, MgCl_2_, ATP and CoA, as well as the enzymes LlALDH1, Ll4CL2 and LlCHS1; (e) Reaction system 2 without Ll4CL2; (f) Reaction system 3 without Ll4CL2; (g) Reaction system 3 without LlALDH1; (h) Reaction system 3 without LlALDH1 and Ll4CL2; (i) Whole reaction system 4: including the substrates dihydro-pCAld and MCoA, cofactors NADP^+^ and CoA, and the enzymes LlCCR and LlCHS1; (j) Reaction system 4 without LlCCR; (k) Potential biosynthetic step of phloretin as well as the required enzymes, substrates, and cofactors. Peak 1, dihydro-pCAci; peak 2, dihydro-pCAld; peak 3, dihydro-CTAL (dihydro-*p*-coumaroyl triacetic acid lactone); peak 4, dihydro-BNY (dihydro-bisnoryangonin); peak 5, phloretin.

In order to identify the potential precursors and enzymes involved in the synthesis of phloretin in *L. litseifolius*, four *in vitro* reaction systems were constructed and the final products were monitored. The results showed that phloretin (*m*/*z* 273) was produced along with two derailment products dihydro-bisnoryangonin (dihydro-BNY) (*m*/*z* 231) and dihydro-*p*-coumaroyl triacetic acid lactone (dihydro-CTAL) (*m*/*z* 273) in System 1 in which the dihydro-pCCoA and MCoA were catalyzed by the recombinant LlCHS1 (Fig. 5a and b). These byproducts were commonly observed in CHS-catalyzed *in vitro* reactions [42]. This indicated that dihydro-pCCoA could serve as a direct precursor for phloretin synthesis. In System 2, phloretin and the two byproducts could be synthesized from dihydro-pCAci and MCoA with combinatorial catalysis of recombinant Ll4CL2 and LlCHS1 and help of cofactors (MgCl_2_, ATP, and CoA), (Fig. 5c), but could not without the Ll4CL2 (Fig. 5e). This suggested that dihydro-pCAci was first converted to dihydro-pCCoA under the action of Ll4CL2, and then be catalyzed by LlCHS1 to produce phloretin. In System 3, phloretin and two byproducts could also be biosynthesized from dihydro-pCAld and MCoA with the combined action of recombinant LlALDH1, Ll4CL2, LlCHS1 enzymes and help of corresponding cofactors (NAD^+^, MgCl_2_, ATP, CoA) (Fig. 5d), but could not without LlALDH1 or Ll4CL2 (Fig. 5f–h). Clearly, dihydro-pCAld could be successively converted to dihydro-pCAci, dihydro-pCCoA, and phloretin by catalysis of the LlALDH1, Ll4CL2 and LlCHS1 step by step. In System 4, phloretin and the two byproducts could be generated from dihydro-pCAld and MCoA with the action of LlCCR and LlCHS1 as well as the help of corresponding cofactors (NADP^+^ and CoA) but could not without LlCCR (Fig. 5i and j). This indicated that dihydro-pCAld could be continuously catalyzed by LlCCR and LlCHS1 to produce phloretin; furthermore, LlCCR played a role in catalyzing the oxidation of aldehyde to acid. According to results of the four reaction systems, dihydro-pCAld could be converted to phloretin through two ways, i.e. through successive catalysis of LlALDH1, Ll4CL2, and LlCHS1, alternatively through catalysis of LlCCR and LlCHS1(Fig. 5k).

**Figure 6:**
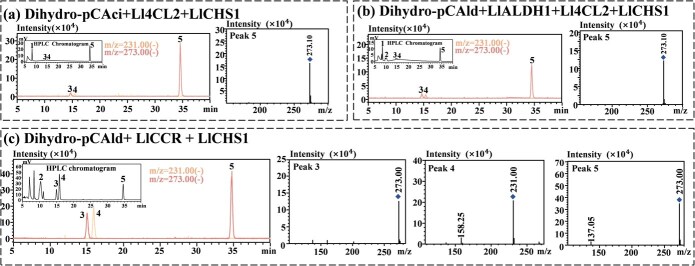
High yield of phloretin from dihydro-*p*-coumaraldehyde (dihydro-pCAld) or dihydro-*p*-cinnamic acid (dihydro-pCAci) catalyzed by LlALDH1, Ll4CL2 and LlCHS1 or LlCCR and LlCHS1 in reaction solutions without cofactor CoA. (a-c) HPLC chromatograms, extracted ion chromatograms (EICs), and MS spectra of the catalytic products in different reaction systems. (a) Reaction system 2 without CoA (including the substrates dihydro-pCAci and malonyl-CoA (MCoA), cofactors MgCl_2_ and ATP, as well as the enzymes Ll4CL2 and LlCHS1). (b) Reaction system 3 without CoA (including the substrates dihydro-pCAld and MCoA, cofactors NAD^+^, MgCl_2_ and ATP, as well as the enzymes LlALDH1, Ll4CL2 and LlCHS1); (c) Reaction system 4 without CoA (including the substrates dihydro-pCAld and MCoA, and cofactor NADP^+^, and the enzymes LlCCR and LlCHS1). Peak 1, dihydro-pCAci; peak 2, dihydro-pCAld; peak 3, dihydro-CTAL (dihydro-*p*-coumaroyl triacetic acid lactone); peak 4, dihydro-BNY (dihydro-bisnoryangonin); peak 5, phloretin.

Interestingly, when the cofactor CoA was removed from Systems 2 to 4 but other ingredients kept unchanged, not only the concentration of the byproducts was significantly reduced but also the yield of phloretin was dramatically increased (Fig. 6). This phenomenon implied that high concentration of CoA in the reaction system would remarkably inhibit the activity of LlCHS1 since four molecules of CoA would be released during the LlCHS1-catalyzed condensation of one molecule of dihydro-pCCoA and three molecules of MCoA, although the cofactor CoA was required for the Ll4CL2/LlCCR-catalyzed synthesis of dihydro-pCCoA from dihydro-pCAci.

### The PGTs contribute to glycosylation of phloretin

**Figure 7:**
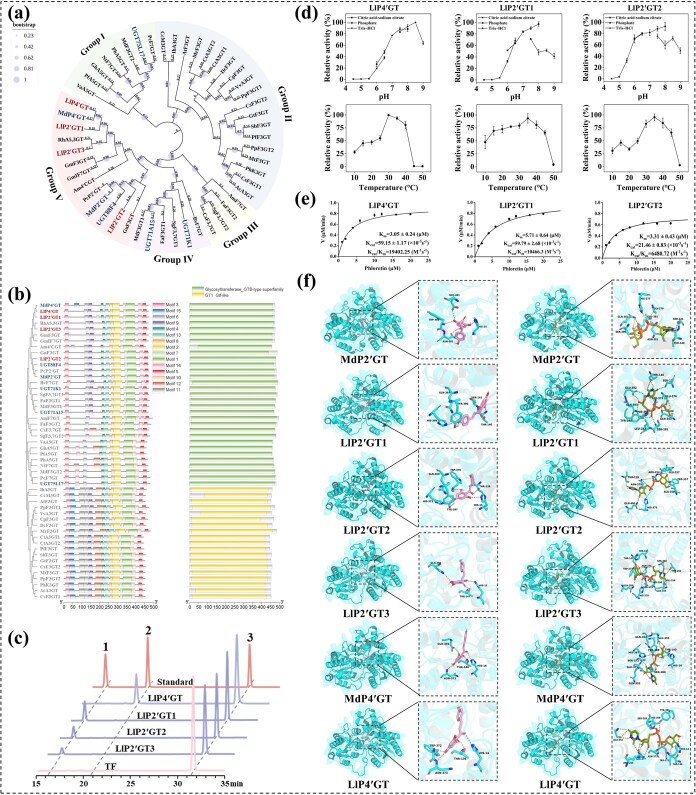
Biological characters of the phloretin glucosyltransferase (PGTs) from *Lithocarpus litseifolius*. (a) Phylogenetic analysis of glucosyltransferases (GTs) from *L. litseifolius* and other species. Information of all the GTs was listed in Table S5. The bootstrap values were indicated by dots in different sizes, and the values on lines represented branch lengths. (b) The conserved motifs identified by MEME Suite and the conserved domains identified by NCBI Batch CD-search in GTs from *L. litseifolius* and other species. (c) Product detection in reactions catalyzed by recombinant PGTs from *L. litseifolius* using phloretin as the substrate. The expressed empty vector protein (TF) was used as the control. The reactions were performed at 30°C and pH 7.5 for 15 min and terminated by addition of trifluoroacetic acid (TFA). (d) The effect of temperature and pH value on recombinant LlP4′GT, LlP2′GT1, and LlP2′GT2. Data were presented as mean ± SD of three repetitions. (e) Kinetic properties of recombinant LlP4′GT, LlP2′GT1, and LlP2′GT2 toward phloretin. The reactions were performed under optimal conditions and terminated by addition of TFA. Data were presented as mean ± SD of three repetitions. Kinetic parameters were estimated after fitting the data to Michaelis–Menten equation. (f) Molecular docking analysis of MdP2′GT, LlP2′GT1, LlP2′GT2, LlP2′GT3, MdP4′GT, and LlP4′GT using phloretin and UDP-glucose as sugar acceptor and donor. 3D models of proteins were predicted by AlphaFold 3. Carbon atoms in phloretin and UDP-glucose were marked in pink and green, respectively. N, O and H atoms were marked in blue, red and white, respectively. Peak 1, phlorizin; peak 2, trilobatin; peak 3, phloretin.

Phloretin 2′-O-glucosyltransferase (P2′GT) and phloretin 4′-O-glucosyltransferase (P4′GT) are responsible for the synthesis of phlorizin and trilobatin, respectively. In our study, four candidate *PGT* genes (*LlP4′GT*, *LlP2′GT1 ~ 3*) from the UGT88 family were cloned from *L. litseifolius*. According to the deduced amino acid sequences, the phylogenetic tree was constructed and shown in Fig. 7a. LlP4′GT and LlP2′GT1 ~ 3 from *L. litseifolius* and 3 PGTs from *M. domestica* were grouped together in Group V. LlP4′GT and LlP2′GT1 were closely related to MdP4′GT (UGT88A32), and LlP2′GT2 was clustered with MdP2′GT (UGT88F1), while LlP2′GT3 showed a close relationship to RhA5,3GT, an anthocyanidin 5,3-O-glucosyltransferase from *Rosa hybrida*. Other PGTs reported to catalyze the glycosidation of phloretin from *M. domestica* were grouped with GTs related to the glycosidation of other flavonoids. Fifteen conserved motifs and two conserved domains were identified in GTs from *L. litseifolius* and other species (Fig. 7b). Motif 1 was included in C-terminal of all the GTs and contained a plant secondary product glycosyltransferase box (PSPG box), which was reported to be involved in binding donor sugars [43]. GTs from Group II contained the most motifs of 14, with motif 15 missing but present in other four groups, while GTs from other groups had multiple missing motifs. As a result, LlP4′GT and LlP2′GT1 ~ 3 were all verified to catalyze the glycosidation of phloretin (Fig. 7c). The optimal conditions and enzyme kinetics of LlP4′GT and LlP2′GT1 ~ 2 were analyzed, except LlP2′GT3 due to its low activity. LlP4′GT showed the highest enzyme activity at pH 8.5 in a 50 mM Tris–HCl buffer and at 30°C, while LlP2′GT1 and LlP2′GT2 were found to be most active at pH 8.0 in a 50 mM phosphate buffer and at 35°C (Fig. 7d). LlP4′GT1 demonstrated the highest affinity for phloretin with a lowest *K*_m_ value (3.05 μM), followed by LlP2′GT2 (3.31 μM) and LlP2′GT1 (5.71 μM). As a result, LlP4′GT (19402.25 M^−1^⋅s^−1^) displayed the highest catalytic efficiency for phloretin, followed by LlP2′GT1 (10466.27 M^−1^⋅s^−1^) and LlP2′GT2 (6480.72 M^−1^⋅s^−1^) (Fig. 7e). According to the result of molecular docking (Fig. 7f), several residues were found to simultaneously interact with phloretin and uridine-5′-diphospho-d-glucose (UDP-Glc) in the tested PGTs, including His-14 in motif 3, Thr-136 and Ser-137 in motif 7, His-369, Trp-372, Asn-373, Tyr-391, Glu-393, and Gln-394 in motif 1 (PSPG box). Among these residues, His-14, His-369, Trp-372, Asn-373, and Gln-394 were highly conserved in all GTs, while Thr-136, Ser-137, Tyr-391, Glu-393 were diverse and less conserved, indicating an important role of the latter in catalyzing glycosyl-transfer toward phloretin.

### DHC accumulation is modulated by expression pattern of the relevant genes

**Figure 8:**
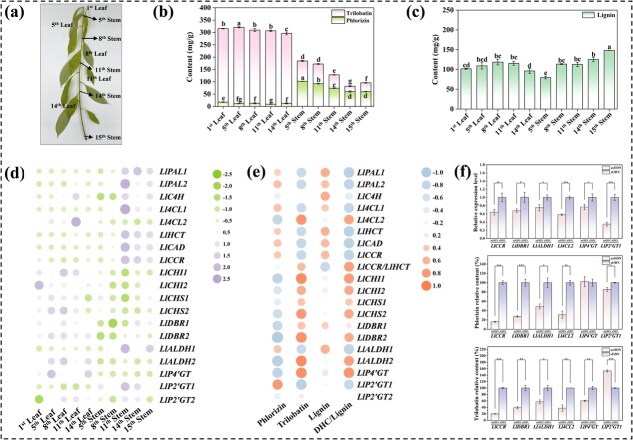
Relationship between content of metabolites and expression levels of the relevant genes in the newly born shoots of *Lithocarpus litseifolius*. (a) Leaves and stems collected from different positions on the shoots of *L. litseifolius.* (b, c) Content of DHCs (b) and lignin (c) in different samples. Data were presented as mean ± SD of three repetitions. Different letters on the bar indicated significant differences (*P* < 0.05). (d) Expression patterns of relevant genes in leaves and stems. Heat map was drawn by the TBtools after the data was normalized. Three biological replicates were carried out for each gene. (e) The correlations between contents of metabolites and expression levels of relevant genes. Heat map was drawn by the TBtools. (f) Effect of antisense oligodeoxyribonucleotides (asODN) treatment on relative gene expression related to DHC biosynthesis and DHC content. *L. litseifolius* tender shoots were immersed in 20 μM asODN solutions and incubated at 25°C with 12 h light/12 h dark for 24 h. The relative expression level was calculated according to 2^-△△CT^ using corresponding sense oligodeoxyribonucleotides (sODN) treatment of each gene as control, and the relative content of the DHCs was calculated using corresponding metabolite level in sODN treatment as 100%. After treatment, the shoots were subjected to gene expression and DHC content analysis. Each treatment was conducted in three biological replicates. Statistical analysis was conducted by Origin 2024 using Bonferroni. ^*^P < 0.05, ^**^P < 0.01, ^***^P < 0.001. Genes from L. litseifolius: *PAL*, *phenylalanine ammonia lyase*; *C4H*, *cinnamate 4-hydroxylase*; *4CL*, *4-coumaroyl: CoA ligase*; *CCR*, *cinnamoyl CoA reductase*; *HCT*, *hydroxycinnamoyl-CoA:shikimate/quinate hydroxycinnamoyl transferase*; *CAD*, *cinnamyl alcohol dehydrogenase*; *CHS*, *chalcone synthase*; *CHI*, *chalcone synthase*; *DBR*, *double bond reductase*; *ALDH*, *aldehyde dehydrogenase*; *P4′GT*, *phloretin 4′-O-glucosyltransferase*; *P2′GT*, *phloretin 2′O-glucosyltransferase*.

In order to clarify the accumulation mechanism of DHCs in *L. litseifolius*, the content of DHCs and lignin in leaves and stem segments at different positions on tender shoots as well as their correlation with expression pattern of the related genes were comparatively studied (Fig. 8a–e), since DHCs and lignin were both present with high content in shoots and shared the upstream metabolic pathway. Phlorizin and trilobatin were the dominant forms of DHC, while phloretin was barely detectable, and two distinctive accumulation patterns of trilobatin and phlorizin were observed in the leaves and stems (Fig. 8b). DHC biosynthesis was greatly activated in the newly expanded leaves, marked by an extremely high level of trilobatin (28.36%–30.92%, DW) and a very low content of phlorizin (0.92%–1.79%, DW), while phlorizin accounted for a higher proportion (53.52%–73.29%) of the total DHCs in stems although content of total DHCs (9.57%–18.45%, DW) was much lower than the corresponding leaves. In addition, content of total DHCs exhibited a trend of first slight rising and then falling from the first leaf to the 14th leaf down the apical bud, but decreased significantly from the fifth stem segment to the 15th stem segment. As a result, a high level of trilobatin was accumulated in tender leaves, while more phlorizin was synthesized in stems and the total DHCs decreased with increased maturity of stem. Content of lignin followed a trend of first slight increase and then decrease from the first leaf to the 14th leaf down the apical bud, but significantly increased from the fifth stem segment (7.95%, DW) to the 15th stem segment (14.85%, DW) of the shoot (Fig. 8c). Clearly, high DHC content but relatively low lignin content was observed in the tender leaves; while continuously increased lignin content but decreased DHC content were witnessed in the stems, indicating a competitive fluctuation between these two metabolites occurred in distinctive modes during development of leaf and stem.

The transcript profiles of some genes involved in phenylpropanoid, lignin, flavonoid, and DHC pathways were measured, including *phenylalanine ammonia lyase* (*LlPAL1 ~ 2*), *cinnamate 4-hydroxylase* (*LlC4H*), *Ll4CL1 ~ 2*, *LlCCR*, *hydroxycinnamoyl-CoA:shikimate/quinate hydroxycinnamoyl transferase* (*LlHCT*), *LlCAD*, *LlDBR1* ~ *2*, *LlALDH1 ~ 2*, *LlCHS1 ~ 2*, *LlCHI1 ~ 2*, *LlP4′GT*, *LlP2′GT1* ~ *2*. These genes could be divided into two major groups according to the expression patterns, *LlDBR1 ~ 2*, *LlALDH2*, *Ll4CL2*, *LlCHS1 ~ 2*, *LlCHI1 ~ 2* and *LlP4′GT* were highly expressed in leaves and mainly exhibited the trends of first increase and then decrease or continuous decrease from the first leaf to the 14th leaf, while *LlPAL1 ~ 2*, *LlC4H*, *Ll4CL1*, *LlCCR*, *LlALDH1*, *LlHCT*, *LlCAD*, and *LlP2′GT* were abundantly expressed in stem and primarily followed a rule of first rising and then falling along with the maturity of stem (Fig. 8d). The correlations between expression of the above genes and the content of metabolites were shown in Fig. 8e. The transcript abundances of *LlDBR1* ~ *2*, *LlALDH2*, *Ll4CL2*, *LlCHS1 ~ 2*, *LlCHI1 ~ 2*, and *LlP4′GT* were positively correlated with trilobatin content, while *LlP2′GT1* expression was positively correlated with phlorizin content. Meanwhile, the expression levels of *LlPAL1 ~ 2*, *LlC4H*, *Ll4CL1*, *LlCCR*, *LlHCT*, and *LlCAD* were positively correlated with lignin content. Interestingly, the ratio DHC/lignin was correlated positively with ratio *LlCCR*/*LlHCT*, besides with the expression of *Ll4CL2*, *LlCHI1 ~ 2*, *LlCHS2*, *LlDBR2*, *LlALDH2*, and *LlP4′GT*, indicating metabolic flux control for biosynthesis of DHCs and lignin was probably modulated by the transcript activity of *LlCCR* vs *LlHCT* which encoded the enzymes responsible for the first step of the DHC and lignin pathways, respectively, although the *LlCCR* was also involved in lignin pathway. In addition, *LlP4′GT* and *LlP2′GT1* were highly expressed in the leaves and stems, respectively, which was in line with the trilobatin and phlorizin profiles in these tissues. Therefore, *LlP4′GT* and *LlP2′GT1* transcript abundance might also attract much more metabolic flux into the DHC pathway.

asODN treatment can efficiently silence the target gene and is an important technology for gene function verification in plants difficult to be transformed, which has been well applied in function analysis of *UGTs* in tea plants [44–49]. In order to confirm the *in vivo* function of the genes involved in DHC synthesis, *L. litseifolius* tender shoots with newly sprouting three leaves were harvested and treated with asODNs targeting the *LlCCR, LlDBR1, LlALDH1, Ll4CL2, LlP4′GT,* and *LlP2′GT1*. Expression levels of the target genes in asODN-treated shoots were significantly lower than those in corresponding sODN-treated samples (Fig. 8f). Meanwhile, phlorizin contents in shoots treated with asODNs decreased significantly compared to those treated with sODNs, except the asODN treatment targeting to *LlP4′GT*, which contributed to trilobatin biosynthesis (Fig. 8f). Similarly, trilobatin content decreased significantly in shoots treated with asODNs, except the asODN treatment targeting *LlP2′GT1* in which trilobatin content increased significantly. The results indicated that biosynthesis of phloretin or trilobatin could be significantly inhibited when *LlCCR, LlDBR1, LlALDH1, Ll4CL2, LlP4′GT,* or *LlP2′GT1* expression were suppressed by asODNs, confirming their significant roles in DHC biosynthesis.

## Discussion

### LlCCR is a multifunctional enzyme

CCR is well recognized for its participation in the biosynthesis of monolignols, namely *p*-coumaryl, coniferyl, and sinapyl alcohols, which corresponds to the hydroxyphenyl (H), guaiacyl (G), and syringyl (S) lignin building blocks, respectively [50]. Most CCRs preferentially catalyze the FCoA, contributing to the production of G- and S-lignins. In contrast, they exhibit low activity toward pCCoA, resulting in a low level of H-lignin, typically less than 5% [51]. In some species, two or more *bona fide* CCRs have been identified. The CCRs involved in lignin biosynthesis were highly expressed in stems, such as *AtCCR1* [52], *PvCCR1* [53], and *MtCCR1* [54]. Conversely, the expression levels of *AtCCR2* and *PvCCR2* were much lower than corresponding *CCR1* counterparts in various tissues and were significantly upregulated upon pathogen infection in leaves, suggesting a role in plant defense. Unlike most CCRs, MtCCR2 showed a preference for caffeoyl CoA (CCoA) and pCCoA, indicating its involvement in a pathway parallel to MtCCR1 for CAld production through methylation of caffeyl aldehyde (CaAld) [54]. In our research, although 12 CCR-encoding genes were cloned and identified from *L. litseifolius*, only LlCCR demonstrated enzymatic activity toward substrates related to lignin biosynthesis. LlCCR exhibited the highest catalytic efficiency toward FCoA, consistent with its well-established role in lignin biosynthesis. However, it also showed relatively high activity toward pCCoA (756.18 M^−1^⋅s^−1^) (Fig. 1d). Given that the H-lignin content in the lignin polymer is usually below 5%, thus, there is reason to believe that LlCCR-catalyzed pCAld may enter an alternative pathway, namely the DHC biosynthesis pathway. Recently, GsCCR, a CCR from *Gloriosa superba*, was reported to participate in the formation of hydroxydihydrocinnamaldehyde in the colchicine biosynthesis pathway for the first time [37], implying CCR might possess multifunction besides lignin synthesis. In addition to the generation of HCAlds, our study also confirmed that LlCCR could catalyze the conversion of dihydro-pCAld to dihydro-pCCoA. Therefore, LlCCR was a multifunctional enzyme that not only participated in lignin biosynthesis but also played a crucial role in DHC biosynthesis by catalyzing the reduction of pCCoA to pCAld and the oxidation of dihydro-pCAld to dihydro-pCCoA (Fig. 1b–d and Fig. 6c). The importance of LlCCR for DHC biosynthesis was also validated through gene silencing test in which the DHC content was significantly downregulated in asODN-treated shoots (Fig. 8f).

**Figure 9:**
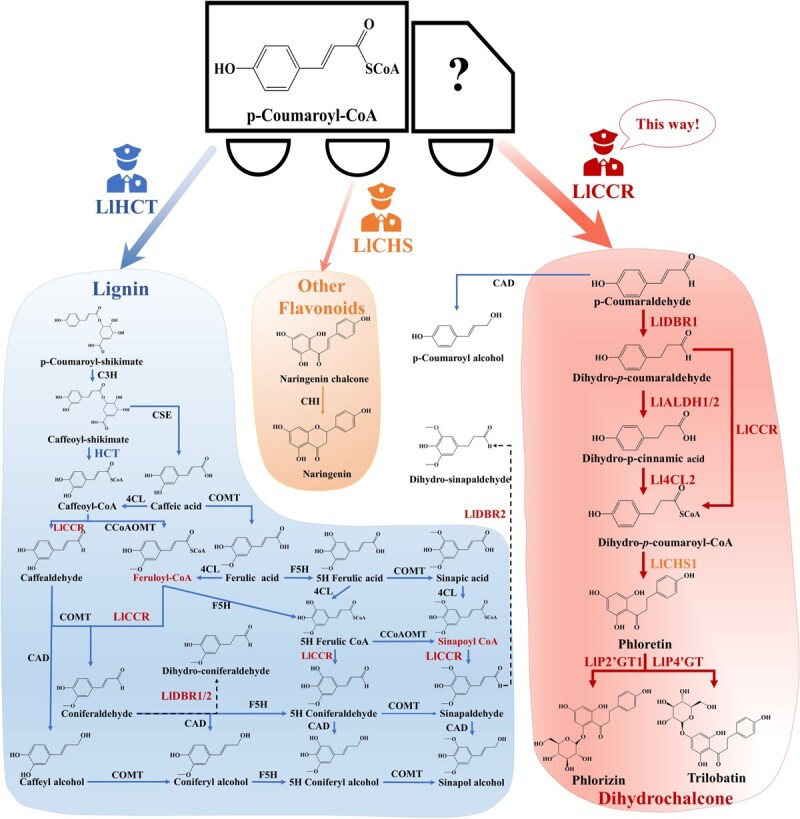
Metabolic flux regulation downstream of p-coumaroyl-CoA in *Lithocarpus litseifolius*. Metabolic flux was mainly pulled and driven into the DHC pathway through highly expressed *LlCCR* and high catalytic efficiency of phloretin glucosyltransferase in tender leaves, and primarily entered into lignin pathway through highly expressed *LlHCT* in growing stems. The flux was indicated by the line width of the arrows. HCT, hydroxycinnamoyl-CoA:shikimate/quinate hydroxycinnamoyl transferase; C3H, coumarate 3 hydroxylase; CSE, caffeoyl shikimate esterase; 4CL, 4-coumaric acid:CoA ligase; CCoAOMT, caffeoyl CoA O-methyltransferase; F5H, ferulate 5-hydroxylase; COMT, caffeate O-methyltransferase; CAD, cinnamyl alcohol dehydrogenase; CHS, chalcone synthase; CHI, chalcone isomerase; CCR, cinnamoyl-CoA reductase; DBR, double-bond reductase; ALDH, aldehyde dehydrogenase; P2’GT, phloretin 2′-O glucosyltransferase; P4'GT, phloretin 4′-O-glucosyltransferase.

*LlCCR* was found to be highly expressed in the stems, along with *LlHCT* and *LlCAD* which worked upstream and downstream of *LlCCR* in lignin biosynthesis, respectively. It took decades to uncover that the conversion of pCCoA to CCoA involves multiple steps (Fig. 9). This process includes the conversion of pCCoA to its shikimate esters by HCT, followed by hydroxylation by *p*-coumarate 3-hydroxylase (C3H) to form caffeoyl-shikimate, which then proceeds through further steps to generate CCoA [55]. As the initial enzymes in the lignin and DHC pathways, respectively, LlHCT and LlCCR may determine the activity of these two pathways. Our study showed that in lignifying stems, the expression levels of *LlCCR* and *LlHCT* were quite similar. However, in tender leaves and the upper stems where DHCs were abundantly synthesized, the expression of *LlCCR* was significantly higher than that of *LlHCT* (Fig. 8d)*.* This suggested that LlCCR and LlHCT might compete for the same substrate pCCoA and direct it into the DHC and lignin pathways, respectively. High-level expression of *LlHCT* would efficiently channel pCCoA into lignin pathway and contribute to FCoA formation from pCCoA in stems. In contrast, low transcript abundance of *LlHCT* would restrict the conversion of pCCoA to FCoA. The high remaining level of pCCoA combined with relatively high *LlCCR* expression, would jointly accelerate the conversion of pCCoA to pCAld, leading to more pCCoA entering the DHC pathway in tender leaves. Interestingly, genes in the phenylpropanoid pathway, including *LlPAL1*, *LlC4H,* and *Ll4CL1*, were strongly activated in the bottom stems but not in tender leaves (Fig. 8d). This indicated that lignin biosynthesis required a large metabolic flux and strongly stimulated the upstream phenylpropanoid genes to produce more pCCoA. Therefore, it was reasonable that as a participant in both the lignin and DHC pathways, *LlCCR* was expressed at a high level only in the lignifying stems but transcribed at a relatively low level in tender leaves with active DHC biosynthesis. The expression ratio of the *LlCCR* to *LlHCT* (*LlCCR*/*LlHCT*) may have a greater impact on the relative activity of DHC vs lignin synthesis than the transcript abundance of *LlCCR* alone (Fig. 8e), although this ratio also needs further verification. The HCT and CHS are the first enzymes of the lignin and flavonoid branches downstream of phenylpropanoid metabolism pathway and can both catalyze pCCoA and direct the metabolic flux into the two branches, respectively [56]. Based on this study, multifunctional LlCCR can also be regarded as one of the key enzymes at the branch point, along with HCT and CHS, to shunt the pCCoA metabolic flux and initiate the DHC biosynthesis pathway (Fig. 9). These findings lay a theoretical framework for the potential *in vitro* synthesis regulation of DHCs in *L. litseifolius*, but the factual regulation of *in vivo* synthesis of DHCs still requires further verification, especially empirical evidence from transgenic study.

### Reduction of the α,β-double bond in pCCoA needs a multistep reaction

Several plant-originated DBRs had been extensively investigated for their ability to catalyze the α,β-unsaturated carbonyl compounds [16, 28, 31]. This adjacent carbonyl group attracts electron density from the double bond, rendering it more electrophilic [57]. To elucidate the physiological functions of DBRs, various potential substrates were initially screened, and many yielded positive results. However, the lack of additional physiological evidence has made it challenging to identify their natural substrates and understand *in vivo* physiological significance. Among the tested substrates, the ability of some DBRs to reduce the α,β-double bond of phenylpropanal substrates, especially pCAld, CAld, and SAld, has been noticed and studied. AtDBR1, also known as P1-ZCr, was first identified to exhibit high activity toward the toxic substrate 4-hydroxy-(2E)-nonenal (HNE), and was inferred to function in stress defense [23]. However, subsequent research found that AtDBR1 showed much higher catalytic efficiency for pCAld (5360 M^−1^⋅s^−1^) and CAld (1060 M^−1^⋅s^−1^) than the previously proposed substrate HNE (600 M^−1^⋅s^−1^) [31]. Two DBRs from *P. appendiculatum* (PaDBRs) were tested with a series of HCAlds, and both showed higher activity toward pCAld and CaAld compared to CAld, 5-hydroxyconiferyl aldehyde (5HCAld) and SAld [33]. Despite the high enzyme activity of these DBRs toward HCAlds, a clear conclusion about the general pathway involving DBRs remains elusive. Recent reports had shown that DBR participates in the biosynthesis of a colchicine precursor by efficiently converting pCAld into dihydro-pCAld with a *K_cat_/K_m_* value of 3166.13 M^−1^⋅s^−1^ [29, 37].

The conventional model of DHC biosynthesis focused on identifying DBRs capable of catalyzing the reduction of the α,β-double bond in pCCoA, as CoA thioesters were generally regarded as the activated forms of the corresponding hydroxycinnamic acids [10]. However, this pathway may not always be favored in plants. It had been reported that the formation of dihydrodehydrodiconiferyl alcohol from dehydrodiconiferyl alcohol involved a three-step process and required conversion to the corresponding aldehyde before reduction by PtPPDBR [26]. Based on our results, pCAld was more likely to be the direct substrate of LlDBR rather than pCCoA, and it might be produced in a manner analogous to the biosynthesis of monolignols by CCR, as previously proposed by another study [37]. In this study, two identified LlDBRs showed activity toward HCAlds with different substrate preferences. LlDBR1 and LlDBR2 showed identical activity toward CAld but remarkably distinct activity toward pCAld and SAld. LlDBR1 possessed the highest *K_cat_* values toward pCAld, as 6.6 times high as toward SAld, while LlDBR2 exhibited a *K_cat_* value for SAld as 28.9 times high as for pCAld (Fig. 2c–e). These results suggested that LlDBR1 catalyzed the reduction of the α,β-double bond in pCAld rather than in pCCoA, while LlDBR2 might play a role in the synthesis of syringyl lactic acid hexoside in lignin biosynthesis [58]. Gene silencing test confirmed that AsODN treatment against *LlDBR1* significantly inhibited the DHC biosynthesis (Fig. 8f). Combining these together, the LlDBR1 was involved in DHC biosynthesis, especially at the conversion step from pCAld to dihydro-pCAld. Clearly, the reduction of the α,β-double bond in pCCoA required at least two steps: converting pCCoA into pCAld and then reducing the α,β-double bond.

### Successive catalysis of ALDHs and 4CLs or independent catalysis of CCR are required for formation of dihydro-pCCoA

Currently, the functions of most *ALDH* genes remain largely enigmatic, particularly the substrate range catalyzed by their encoded proteins [59]. For instance, *A. thaliana* AtALDH3H1 and AtALDH3I1 were capable of catalyzing the oxidation of saturated fatty aldehydes, including propionaldehyde, nonanal, octanal, and dodecanal. Notably, their catalytic efficiency increased with the elongation of the carbon chain. However, they showed weak catalytic activity toward unsaturated fatty aldehydes and demonstrated no catalytic activity against aromatic aldehydes [60]. In addition, *A. thaliana* AtALDH2C4, as well as *Oryza sativa* OsALDH2C2 and OsALDH2C3, were associated with the biosynthesis of FAci and SAci which served as cell wall cross-linking agents [61, 62], implying members from the ALDH2 subfamily can catalyze HCAld to the corresponding acids. In this study, three ALDHs able to catalyze HCAld were identified from *L. litseifolius*, namely LlALDH3 from the ALDH2 subfamily as well as LlALDH1 and LlALDH2 from the ALDH3 subfamily (Fig. 3a–c). Among them, LlALDH1 and LlALDH2 exhibited higher catalytic activity toward dihydro-pCAld. The ALDH2 and ALDH3 subfamilies had a distant phylogenetic relationship on the evolutionary tree and the discovery of ALDH3 subfamily members capable of catalyzing HCAld represented a novel finding. Enzyme kinetics analysis revealed that both LlALDH1 and LlALDH2 preferred dihydro-pCAld, but LlALDH1 had stronger catalytic activity toward dihydro-pCAld, pCAld, CAld, and SAld compared to LlALDH2 (Fig. 3d). In gene silencing test, the DHC content decreased significantly in tender shoots treated with AsODN against *LlALDH1* (Fig. 8f). Based on the results of enzyme kinetics and gene expression, it could be inferred that LlALDH1 and LlALDH2 may be both involved in DHC pathway in *L. litseifolius*, although expressions of the *LlALDH1* and *LlALDH2* were highly activated in stems and leaves, respectively.

It had been reported that many plants have two types of 4CLs with different structures and functions. Class I 4CL is potentially involved in lignin metabolism through two primary mechanisms. It can generate pCCoA to supply the precursor for HCT, or produce CCoA to serve as a precursor for caffeoyl CoA O-methyltransferase [63, 64]. Additionally, *Rehmannia glutinosa* Rg4CL1 directed the metabolic flux to acteoside synthesis by catalyzing the generation of CCoA [65]. Class II 4CL, on the other hand, is likely to engage in flavonoid metabolism. For example, overexpression of the *R. glutinosa Rg4CL2* led to a significant increase in flavonoid content. However, the specific biochemical reactions in which this class of 4CL participates within the flavonoid synthesis pathway remain unclear [65]. Evolutionary analysis showed that Ll4CL2 clustered in the dicot clade II together with Class II At4CL3, while Ll4CL1 and Ll4CL3 clustered in dicot clade I with Class I At4CL1, At4CL2, and At4CL4. Similar to most plant 4CLs, Ll4CL1 and Ll4CL2 had no activity toward SAci, but could catalyze pCAci, FAci, and dihydro-pCAci. Tests also showed that *Ll4CL1* and *Ll4CL2* were highly expressed in the stems and in leaves, respectively. These findings suggested that Ll4CL1 may be the major contributor to lignin synthesis, while Ll4CL2 may play a dominant role in DHC synthesis. In future research, enzyme kinetic analysis of Ll4CL1 and Ll4CL2 toward substrates such as pCAci, FAci, and dihydro-pCAci, and caffeic acid should be further tested in order to clarify their substrate preferences.

This study confirmed that dihydro-pCAci could be converted into dihydro-pCAld by action of LlALDH1, dihydro-pCCoA could be generated from dihydro-pCAci through catalysis of Ll4CL2 or directly from dihydro-pCAld through catalysis of LlCCR, and then phloretin was further generated under the action of LlCHS1. Thus, phloretin could be achieved through successive catalysis of LlALDH1, Ll4CL2, and LlCHS1 or by combined action of LlCCR and LlCHS1 in turn from direct precursor dihydro-pCAld which was generated from pCCoA through catalysis of LlCCR and LlDBR (Figs 5 and 9). It was worth mentioning that CHS exhibited catalytic promiscuity. When *Escherichia coli* or yeast-derived CHS catalyzed p-CCoA *in vitro*, it frequently generated the secondary condensation derailment product BNY and the tertiary condensation derailment product CTAL. Similarly, when dihydro-pCCoA was used as the substrate, the derailment products were dihydro-BNY and dihydro-CTAL [21, 66]. This study discovered that when CoA was removed from the reaction system using dihydro-pCAld or dihydro-pCAci as the starting substrate, the two byproducts were virtually undetectable in the product, and the yield of phloretin increased substantially (Fig. 6). This observation implied that Ll4CL2 and LlCHS1 might form a complex, enabling them to jointly catalyze dihydro-pCAci and to efficiently and specifically produce phloretin through CoA sharing. Conversely, the presence of a high concentration of CoA in the system would impede the formation of the Ll4CL2 and LlCHS1 complex, resulting in an increase in byproducts.

### Phloretin-specific PGTs serve as a strong pump activating the DHC pathway

Glycosylation can significantly impact the solubility, stability, biological activities, color, and taste of flavonoids [67]. In most *Malus* species, phlorizin was the primary and often the sole form of DHC, while a few species also contained trilobatin and sieboldin (3-hydroxyphloretin 4′-O-glucoside) [1]. Several GTs from the *Malus* species involved in the *in vitro* glycosylation of phloretin had been reported, including UGT88F1 [12], UGT88F4 [68], UGT71K1 [69], UGT71A15 [69], UGT75L17 [70], and UGT88A32 [13]. Among them, UGT75L17 and UGT88A32 could produce trilobatin, while others catalyzed the formation of phlorizin. Notably, UGT71A15 also produced a small amount of trilobatin and phloretin-4-O-glucoside. UGT71K1, UGT71A15, and UGT75L17 showed broad substrate acceptance, such as kaempferol and quercetin, suggesting their involvement in the glycosylation of other flavonoids besides phloretin. In contrast, UGT88F1 and its homolog in *Pyrus communis*, UGT88F2, exhibited highly specific activity toward phloretin [12, 69]. In *L. litseifolius*, Zhang *et al*. [71] characterized 3 PGTs that used phloretin as a substrate, Cluster-6439.111627 (P2′GT), Cluster-6439.143031 (P2′GT), and Cluster-6439.98883 (P4′GT), with the former two corresponding to LlP2′GT1 and LlP2′GT2 in this study. The *K*_cat_*/K*_m_ value of Cluster-6439.143031 was 19.7 times high as that of Cluster-6439.98883. In our research, two additional PGTs, LlP2′GT3 and LlP4′GT, were identified to catalyze the formation of phlorizin and trilobatin, respectively. In the phylogenetic tree, all active PGTs were clustered with GTs from the UGT88 family in group V, including the well-recognized MdP2′GT (UGT88F1) and MdP4′GT (UGT88A32). This indicated that UGT88 family may be a significant source of phloretin-specific PGTs. Based on the kinetic properties (*K*_cat_/*K*_m_ values of 19402.25 M^−1^⋅s^−1^ for LlP4′GT and 10466.3 M^−1^⋅s^−1^ for LlP2′GT1 toward phloretin) and expression profiles in this study (Figs 7 and 8), LlP4′GT and LlP2′GT1 were more likely to play a major role *in planta*, strongly driving the accumulation of DHCs.

In domesticated apples, the DHC profile was mainly composed of phlorizin, and its significance for plant development had been extensively studied. In *UGT88F1* knockdown lines, phlorizin accumulation decreased significantly (by 65%–75%), resulting in severe dwarfing, with greatly reduced internode lengths and narrow, spindly leaves [72]. It was also observed that phloretin did not accumulate despite the decrease in phlorizin, indicating that PGTs significantly influenced the overall activity of the DHC pathway. Phlorizin biosynthesis also affected resistance to *Valsa* canker and coordination between carbon and nitrogen accumulation [73, 74]. Transgenic apple plants with reduced phlorizin through silencing *MdPGT1* but restored total DHCs by over-expressing *FcCGT* (a kumquat-derived GT gene encoding the UGT708G1 responsible for synthesis of phloretin 3′,5′-di-C-glycoside) were severely dwarfed and more susceptible to drought stress. These indicated that phlorizin was essential for apple plant development [75]. Measurements showed that in mature leaf and root of *L. litseifolius*, phlorizin was dominant while trilobatin was present in trace amounts (Fig. S6). Similar to most *Malus* species, phlorizin might be a stable storage form of DHC in the roots, mature leaves, and stems of *L. litseifolius*. During shoot development, tender leaves at different positions on the shoot contained high levels of trilobatin but only trace amounts of phlorizin. A continuous decrease in the contents of trilobatin and phlorizin was observed from the top to the bottom of the tender stem (Fig. 8b). This suggested that trilobatin and phlorizin are crucial for the rapid development of tender shoots, and trilobatin is important for leaf development. Phlorizin had been reported to undergo a different degradation process compared to trilobatin and phloretin [76], which might reflect their different physiological functions. Trilobatin and phloretin were susceptible to oxidation and could be converted to seboldin and 3-hydroxy phloretin by polyphenol oxidase before being finally converted to oxidation products. In contrast, phlorizin degraded much more slowly and underwent hydrolysis step by specific β-glucosidases to its aglycone, phloretin, before downstream oxidation. The above research could explain why phlorizin was more stable than trilobatin in mature tissues. However, the physiological role of the extremely high content of trilobatin during shoot development and its degradation fate during maturation remained to be elucidated.

## Materials and methods

### Chemicals

pCCoA, FCoA, SCoA, pCAld, CAld, SAld, dihydro-pCAld, pCAci, dihydro-pCAci, FAci, SAci, phloretin, phlorizin, trilobatin, phenylmethanesulfonylfluoride (PMSF), NADP^+^, NAD^+^, NADPH, CoA, and ATP were purchased from Shanghai Yuanye Biotechnology Co., Ltd (Shanghai, China). Dihydro-coniferyl aldehyde was bought from Accela ChemBio Co., Ltd (Shanghai, China). Isopropyl-β-D-1-thiogalactopyranoside (IPTG), MCoA, trifluoroacetic acid (TFA), UDP-Glc, and formic acid (HPLC grade) were purchased from Shanghai Macklin Biochemical Co., Ltd (Shanghai, China). Acetonitrile (HPLC grade) was obtained from Sigma-Aldrich Corporation (St. Louis, USA). Lysozyme and imidazole were bought from Yeasen Biotech Co. Ltd (Shanghai, China). MilliQ water was prepared by Barnstead™ GenPure™ water system (ThermoFisher Scientific, USA) and used throughout the experiment. Unless otherwise stated, all other reagents were obtained from Sinopharm Chemical Reagent Co., Ltd (Shanghai, China).

### Plant materials

The tender shoots, mature leaves, and roots of *L. litseifolius* were kindly provided by Huzhou Xinya Medicinal Materials Technology Co., Ltd (Zhejiang, China). Tender shoots with 15–17 expanding leaves were plucked and the first leaf (1st leaf), fifth leaf (5th leaf), eighth leaf (8th leaf), eleventh leaf (11th leaf), and fourteenth leaf (14th leaf), as well as their corresponding stem segments (the 5th stem, 8th stem, 11th stem, and 14th stem) and the basal stems (15th stem) were collected. All the collected samples were divided into two parts: one was fixed by microwave at 800 W for 2 min and dried at 80°C to constant weight, and then used for lignin and DHC analysis; another used for gene cloning and quantitative real-time PCR (qPCR) analysis was frozen in liquid nitrogen and stored at −80°C until further use.

### Screening out the candidate genes of CCRs, DBRs, ALDHs, 4CLs, and GTs from *L. litseifolius*

As there was no genome assembly data of *L. litseifolius*, RNA-seq was performed in order to obtain the candidate genes related to DHCs synthesis. Total RNAs were extracted from newly spouted young leaves, fully expanded mature leaves, and old leaves with different composition and level of DHCs using an Easy Plant RNA Kit (Zhejiang Easy-Do Biotech Co. Ltd, Hangzhou, China) according to the manual. Three biological replicates were conducted. Transcriptome sequencing was performed by Shanghai Personal Biotechnology Co., Ltd. The expressed genes were assembled and annotated in seven public databases namely the NCBI non-redundant protein sequences database, NCBI non-redundant nucleotide database, Protein family database, euKaryotic Orthologous Groups, Gene Ontology, Clusters of Orthologous Groups of protein databases and Swiss-Prot protein database.

Using *AtCCR1* (*At1g15950*) [52], *AtDBR* (*At5g16970*) [31], *AtALDH2C4* (*At3g24503*) [61], *At4CL1* (*At1g51680*) [41] and *MdP2′GT* (NM_001328723.2) [12] as the reference sequences, potential candidate genes in *L. litseifolius* were screened out from the transcriptome data by TBtools [77] and deduced into amino acid sequences. The phylogenetic trees were constructed according to the amino acid sequences using the neighbor-joining method with 1000 bootstrap replications in MEGA 11 and refined by iTOL (https://itol.embl.de/login.cgi). The accession information of the CCRs, DBRs, ALDHs, 4CLs, and GTs was listed in Table S1, Table S2, Table S3, Table S4 and Table S5, respectively.

### Cloning of the candidate genes

The cDNA synthesis was performed according to the instructions of the HiScript™ II Q RT SuperMix Kit (Vazyme Biotech Co. Ltd, Nanjing, China) using the extracted total RNAs as templates. Based on the transcriptome information of *L. litseifolius*, the open reading frames (ORFs) of genes *LlCCR*, *LlCCRL1 ~ 11*, *LlDBR1* ~ *6*, *LlALDH1 ~ 6, Ll4CL1 ~ 4, LlCHS1*, *LlP4′GT*, and *LlP2′GT1 ~ 3* were cloned from the cDNA library using the primers in Table S6 and constructed into the expression vector pCold-TF through homologous recombination strategy using ClonExpress II One Step Cloning Kit (Vazyme Biotech Co. Ltd, Nanjing, China) and transformed into *E. coli* (DH5α) for sequence verification. After that, all the cloned genes were deposited in GenBank, and the accession numbers were PQ867195 (*LlCCR*), PQ867203 ~ PQ867213 (*LlCCRL1–11*), PQ867196 (*LlCHS1*), PQ867197 (*LlDBR1*), PQ867198 (*LlDBR2*), PQ867214 (*LlDBR3*), PQ867217 (*LlDBR4*), PQ867216 (*LlDBR5*), PQ867215 (*LlDBR6*), PX215327 (*LlALDH1*), PX215326 (*LlALDH2*), PX215328 (*LlALDH3*), PX21529 (*LlALDH4*), PX21530 (*LlALDH5*), PX215331 (*LlALDH6*), PX215322 (*Ll4CL1*), PX215323 (*Ll4CL2*), PX215324 (*Ll4CL3*), PX215325 (*Ll4CL4*), PQ867201 (*LlP4′GT*), PQ867199 (*LlP2′GT1*), PQ867200 (*LlP2′GT2*), PQ867202 (*LlP2′GT3*).

### Expression and purification of recombinant proteins

The verified constructs and the pCold-TF empty vector were transformed into the *E. coli* strain Rosetta (DE3) (Beijing Biomed Gene Technology Co., Ltd, Beijing, China). The transformed strains were incubated at 37°C and 200 rpm until OD_600_ reached 0.6, followed by a 16-h induction at 15°C by 1 mM IPTG. After centrifugation at 5000 rpm for 10 min at 4°C, the cell pellets were resuspended in the lysis buffer (50 mM Na_2_HPO_4_, 300 mM NaCl, 10 mM imidazole, pH 8.0) containing 1 mg⋅ml^−1^ lysozyme and 1 mM PMSF, and disrupted by sonication (SCIENTZ-IID, Ningbo, China). The lysis product was centrifuged at 15000 rpm for 30 min, and the supernatant was filtered through a 0.22-μm Syringe Filter (Merck & Co. Inc, Rahway, NJ USA) before being applied to the HisSep Ni-NTA Agarose column (Yeasen Biotech Co. Ltd, Shanghai, China) for purification according to the manufacturer’s protocol. The purity of the protein was analyzed using SDS-PAGE (Fig. S1), and the concentration was determined using a BCA Protein Quantification Kit (Yeasen Biotech Co. Ltd, Shanghai, China).

### Enzyme assays

Enzyme activity assays were conducted in reaction mix containing the recombinant protein (~2 mg⋅ml^−1^), substrates, and cofactors, and the mixture was made up to 250 μl by addition of suitable buffer solution. Reactions were generally terminated by the addition of TFA.

pCCoA, FCoA, SCoA were used as substrates for recombinant LlCCR, and LlCCRL1 ~ 11. The reaction mixture contained 200 μM substrate, 200 μM NADPH, and 10 μl of purified protein and the reaction was conducted at 30°C and at pH 6.0 for 30 min. Dihydro-pCAld was also used as the substrate to investigate the reverse reaction of LlCCR. The reaction was conducted with 500 μM NADP^+^, 500 μM CoA, 500 μM dihydro-pCAld, and 10 μl of purified protein at 30°C and pH 8.0 for 30 min. The reactions were terminated either by adding 5 μl of TFA or by adding 11.2 μl of 5 N NaOH and then hydrolyzing at 45°C for 30 min. Hydrolysis with NaOH was performed in order to release the pCAci, FAci, SAci, and dihydro-pCAci for subsequent HPLC analysis as their CoA thioester derivatives (pCCoA, FCoA, SCoA, and dihydro-pCCoA) are difficult to detect directly [10]. pCAld, CAld, and SAld were used as substrates for recombinant LlDBR1 ~ 6 and the enzyme assays were performed with 500 μM substrate, 500 μM NADPH, and 10 μl of purified protein at 30°C and pH 6.0 for 30 min. Dihydro-pCAld, pCAld, CAld, and SAld were used as substrates for recombinant LlALDH1 ~ 6 and the enzyme activities were assayed with 500 μM substrate, 500 μM NAD^+^, and 10 μl of purified protein at 30°C and pH 6.5 for 30 min. pCAci, dihydro-pCAci, FAci, and SAci were used as substrates for recombinant Ll4CL1 ~ 2, and the enzyme assays were conducted with 500 μM substrate, 500 μM CoA, 2.5 mM ATP, 2.5 mM MgCl_2_, and 10 μl of purified protein at 30°C and pH 6.5 for 30 min. Enzyme assays of LlP4′GT, LlP2′GT1 ~ 3, were performed with 100 μM phloretin, 100 μM UDP-Glc, and 2.5 μl of purified protein at 30°C and pH 7.5 for 15 min.

The optimal pH value and temperature tests were determined for LlCCR, LlDBR1 ~ 2, LlALDH1 ~ 2, LlP4′GT, and LlP2′GT1 ~ 2. For LlCCR, pCCoA and dihydro-pCAld were used as substrates. For LlDBR1 ~ 2, pCAld was used as the substrate. For LlALDH1 ~ 2, dihydro-pCAld was used as the substrate. For LlP4′GT, and LlP2′GT1 ~ 2, phloretin and UDP-Glc were used as the substrates. For the pH value tests, 50 mM sodium citrate buffer (pH 4.5–6.5), 50 mM phosphate buffer (pH 6.0–8.0), and 50 mM Tris–HCl buffer (pH 7.5–9.0) were used, and the reactions were performed at 30°C for 10 min. For the optimal temperature tests, the reactions were performed at 10°C to 50°C at intervals of 5°C for 10 min in optimal buffer. After reaction termination, the solutions were centrifuged for 10 min at 12 000 rpm before HPLC analysis. All enzyme assays were conducted in triplicate.

### Enzyme kinetics

Kinetic analysis was conducted at optimal pH value and temperature in a 250-μl reaction system. The kinetic analysis of LlCCR toward pCCoA, FCoA, and SCoA was conducted with 5 μl of protein solution, 200 μM NADPH, and substrates at a range of concentrations for 5 min. For LlDBRs, reaction mixture contained 5 μl of protein solution, 500 μM NADPH, and substrates at a range of concentrations. The reaction time of LlDBR1 and LlDBR2 for substrates pCAld and CAld was 10 min, and that for substrate SAld was 30 and 2.5 min, respectively. The kinetic analysis of LlALDHs toward dihydro-pCAld, pCAld, CAld, and SAld was performed in a series of reaction solutions including 5 μl of protein solution, 500 μM NADP^+^, and substrates at a range of concentrations. The reaction time of LlALDH1 was 5 min for dihydro-pCAld and 10 min for the other substrates, and the reaction time of LlALDH2 was 10 min for dihydro-pCAld and 20 min for the others. The kinetic analysis of LlP2′GT1 ~ 2 and LlP4′GT was performed in reaction mixtures including 2.5 μl of protein solution, 20 μM UDP-Glc, and phloretin from 2.5 to 20 μM for 5 min. After being terminated by the addition of 5 μl of TFA, the reaction solutions were centrifuged for 10 min at 4°C and 12 000 rpm before HPLC analysis. The kinetic parameters (*K*_m_, *V*_max_, *K*_cat_, *K*_cat_/*K*_m_) were calculated though fitting the experiment data to the Michaelis–Menten nonlinear regression curve on the software Origin 2024 (OriginLab, Hamptons, USA). All enzyme assays were conducted in triplicate.

### Phloretin *in vitro* synthesis assays

System 1 (Dihydro-pCCoA + LlCHS1): After mixing the 500-μM dihydro-pCCoA, 500 μM MCoA, and 10 μl of purified LlCHS1 in a 50 mM phosphate buffer at pH 7.5, the reaction was carried out at 30°C for 1 h.

System 2 (Dihydro-pCAci + Ll4CL2 + LlCHS1): The reaction mixture contained 500 μM dihydro-pCAci, 500 μM CoA, 500 μM MCoA, 2.5 mM ATP, 2.5 mM MgCl_2_, and 10 μl of purified LlCHS1 and Ll4CL2. The reaction was carried out in a 50 mM phosphate buffer at pH 7.5 and at 30°C for 1 h. A reaction without recombinant Ll4CL2 was used as the control.

System 3 (Dihydro-pCAld + LlALDH1 + Ll4CL2 + LlCHS1): The reaction mixture contained 500 μM dihydro-pCAld, 500 μM CoA, 500 μM NAD^+^, 500 μM MCoA, 2.5 mM MgCl_2_, 2.5 mM ATP, and 10 μl of purified LlALDH1, Ll4CL2, and LlCHS1. The reaction was conducted in a 50 mM citrate buffer at pH 6.5 and at 30°C for 1 h. Reactions without recombinant Ll4CL2 or LlALDH1 were used as controls.

System 4 (Dihydro-pCAld + LlCCR + LlCHS1): After mixing 500 μM dihydro-pCAld, 500 μM MCoA, 500 μM NADP^+^, 500 μM CoA, and 10 μl of LlCCR and LlCHS1 in a 50 mM citrate buffer at pH 6.5, the reaction was performed at 30°C for 1 h. A reaction without LlCCR was used as the control.

After being terminated by the addition of 5 μl of TFA, the solution was centrifuged for 10 min at 4°C and 12 000 rpm before HPLC-UV-MS analysis. All enzyme assays were conducted in triplicate.

### Molecular docking analysis

3D models of MdP2′GT, MdP4′GT, LlP2′GT1, LlP2′GT2, LlP2′GT3 and LlP4′GT were established on the platform of AlphaFold 3 (https://alphafoldserver.com). UDP-Glc and phloretin were docked into the active site of each enzyme using Autodock Vina [78]. The docking results were visualized using PyMOL (version 2.6.0).

### Extraction of DHCs from sweet tea samples

About 150 mg of dried *L. litseifolius* sample was ground into powder and extracted with 25 ml of a 50% (v/v) ethanol solution at 70°C for 20 min. The extract was cooled down to room temperature, centrifuged at 12 000 rpm and 4°C for 10 min, and then 1 ml of the supernatant was transferred into a 1.5-ml vail before being applied to HPLC analysis. Each sample was performed with three biological replicates.

### Analysis of lignin

The *Kalson* lignin was prepared as described by Beramendi-Orosco *et al.* [79] with some modifications. About 500 mg of *L. litseifolius* powdered sample was soaked with 25 ml of a 95% (v/v) ethanol solution at 70°C for 30 min and the supernatant was discarded after centrifugation at 5000 rpm for 10 min. The pellets were soaked twice as above, then the pellets were mixed with 5 ml of 72% (v/v) H_2_SO_4_ for hydrolysis. After 2 h, the suspension was diluted by 40 ml of water and boiled in a water bath for 4 h. The insoluble residue, namely lignin, was obtained by suction filtration and dried to constant weight, and then the dried lignin was weighted. Lignin content was expressed as a percentage of the dry weight of *L. litseifolius*. Each sample was performed with three biological replicates.

### Expression suppression of relevant genes by asODN treatment

Candidate sequences of asODNs and sense oligodeoxyribonucleotides (sODNs) (Table S7) were designed using RNAfold online software (https://sfold.wadsworth.org/cgi-bin/soligo.pl), and the last three nucleotides at both end of the oligonucleotide were modified with thiophosphorylations for stability. The sODNs were used as control. AsODNs and sODNs were synthesized by Sangon Biotech Co., Ltd (Shanghai, China). *L. litseifolius* tender shoots with three newly grown small leaves were immersed in 1 ml oligonucleotide solution (20 μM in 1/2 Murashige & Skoog medium) in 1.5 ml centrifuge tubes and incubated at 25°C and 12 h light (3000 lux)/12 h dark for 24 h. Three biological replicates were carried out for each gene. After incubation, the first leaves were sampled from each treatment and used for gene expression analysis, and the remaining samples of each treatment were denatured by microwave at 800 W for 1 min, and dried at 80°C for 4 h before HPLC analysis.

### Transcript level analysis of relevant genes

qPCR was conducted to detect the transcript profiles of relevant genes. The reaction mixture was prepared according to the manual of ChamQ SYBR qPCR Master Mix (Vazyme Biotech Co. Ltd, Nanjing, China) with specific primers (Table S8) and qPCR was performed on an ABI StepOnePlus™ real-time PCR detection system (Applied Biosystems, CA, USA). Triplicate reactions were conducted for each gene. Relative expression levels were calculated using the 2^−ΔCt^ method using *β-actin* as the reference gene.

### HPLC-UV-MS analysis

The DHCs in *L. litseifolius* plant samples and in enzyme reaction solution were analyzed by a 20A HPLC equipped with a UV detector and MS detector (Shimadzu Co., Kyoto, Japan). The HPLC conditions were as follows: chromatographic column, Zorbax 5 μm TC-C18(2) (250 × 4.6 mm, Agilent Technologies Inc., CA, USA); injection volume, 10 μl; oven temperature, 35°C; mobile phase A, formic acid/water (0.5/99.5, v/v); mobile phase B, acetonitrile/formic acid (99.5/0.5, v/v); gradient elution, maintaining 22% phase B in early 10 min, and linearly increasing to 35% in the next 20 min, then holding 35% for 10 min, and then shifting back to 22% in the next 5 min; flow rate, 1 ml⋅min^−1^; detecting wavelength, 280 nm.

The substrates and products in the reaction solution catalyzed by LlCCR, LlDBR1 ~ 2, LlALDH1 ~ 3 and Ll4CL1 ~ 2 were monitored under similar conditions as above with some modifications: oven temperature, 43°C; mobile phase A, acetonitrile/formic acid/water (3/0.5/96.5, v/v/v); mobile phase B, acetonitrile/formic acid/water (30/0.5/69.5, v/v/v); gradient elution, the phase B linearly increasing from 35% to 80% in early 35 min and then shifting back to 35% in the next 5 min.

For phloretin identification, electrospray ionization (ESI) was performed in the negative ionization mode with the following parameters: the interface temperature, 350°C; desolvation temperature, 250°C; heat block temperature, 200°C; the nebulizing gas, 1.5 l⋅min^−1^; the drying gas, 15 l⋅min^−1^; the full-scan mode, 100–300 *m*/*z*.

### Statistical analysis

Significant difference analysis was conducted by IBM SPSS Statistics 26 (IBM, Chicago, IL, USA) using one-way ANOVA and Duncan’s post-hoc test and *P* < 0.05 was considered as significant difference. Correlation analysis was performed according to Spearman’s parametric correlation test.

## Supplementary Material

Web_Material_uhag061

## Data Availability

All data related to this research are available in this paper and its supplementary materials published online.
