# Supplementary material for: Study on biosynthesis pathway and accumulation mechanism of the dihydrochalcones in Lithocarpus litseifolius
Source: Hortic Res. 2026 Feb 27;13(6):uhag061. doi: 10.1093/hr/uhag061 (PMC13249509; doi:10.1093/hr/uhag061)
Supplement: Web_Material_uhag061 [file web_material_uhag061.zip › Supplementary files_revision marked.docx]

**Study on biosynthesis pathway and accumulation mechanism of the dihydrochalcones in *Lithocarpus litseifolius***

Yu-Si Yang^1^, Yu-Ke Du^1^, Jia-Li Li^1^, Yong-Kang Wang^1^, Cun-Yu Li^1^, Xin-Qiang Zheng^1^, Jian-Hui Ye^1^, Yue-Rong Liang^1^, Zhou-Tao Fang^1,2,*^, Jian-Liang Lu^1,*^

1. Tea Research Institute, Zhejiang University, Hangzhou (310058), PR China

2. Shaoxing Jianming Tea Industry Co., Ltd, Hangzhou (310058), PR China

Author’s email: YS Yang, 12016069@zju.edu.cn; YK Du, 12416098@zju.edu.cn; JL Li, 22316218@zju.edu.cn; YK Wang, wongykang@zju.edu.cn; CY Li, [3160100115@zju.edu.cn](mailto:3160100115@zju.edu.cn); XQ Zheng, [xqzheng@zju.edu.cn](mailto:xqzheng@zju.edu.cn); JH Ye, jx0515@163.com; YR Liang, [yrliang@zju.edu.cn](mailto:yrliang@zju.edu.cn); ZT Fang, [11916073@zju.edu.cn](mailto:11916073@zju.edu.cn); JL Lu, [jllu@zju.edu.cn](mailto:jllu@zju.edu.cn)

*. Corresponding author.

Supplementary files

**Table S1.** Accession numbers of cinnamoyl-CoA reductases (CCRs) and CCR (CCRL) proteins in the phylogenetic tree.

| Protein | Organism | Family | Accession |
| --- | --- | --- | --- |
| AcCCR1 | *Ananas comosus* | Bromeliaceae | XP_020083039.1 |
| AcCCR2 | *Ananas comosus* | Bromeliaceae | XP_020102291.1 |
| AtCCR1 | *Arabidopsis thaliana* | Brassicaceae | NP_001319013.1 |
| AtCCR2 | *Arabidopsis thaliana* | Brassicaceae | AAG53687.1 |
| AtCCRL1 | *Arabidopsis thaliana* | Brassicaceae | NP_177021.1 |
| AtCCRL2 | *Arabidopsis thaliana* | Brassicaceae | NP_177773.2 |
| AtCCRL3 | *Arabidopsis thaliana* | Brassicaceae | NP_180917.1 |
| AtCCRL4 | *Arabidopsis thaliana* | Brassicaceae | NP_180918.1 |
| AtCCRL5 | *Arabidopsis thaliana* | Brassicaceae | NP_178345.1 |
| AtCCRL6 | *Arabidopsis thaliana* | Brassicaceae | NP_565557.1 |
| AtCCRL7 | *Arabidopsis thaliana* | Brassicaceae | NP_194776.1 |
| BdCCR1 | *Brachypodium distachyon* | Poaceae | XP_003560992.1 |
| BdCCR2 | *Brachypodium distachyon* | Poaceae | XP_003574536.1 |
| BpCCR | *Betula platyphylla* | Betulaceae | AIX92152.1 |
| BvCCR | *Beta vulgaris* | Amaranthaceae | XP_010691595.1 |
| CasiCCR | *Camellia sinensis* | Theaceae | KAF5957199.1 |
| CciCCR | *Corymbia citriodora* | Myrtaceae | KAF8021650.1 |
| CdCCR | *Castanea dentata* | Fagaceae | KAF3973871.1 |
| CiCCR1 | *Carya illinoinensis* | Juglandaceae | KAG2723250.1 |
| CiCCR2 | *Carya illinoinensis* | Juglandaceae | XP_042951286.1 |
| CisCCR | *Citrus sinensis* | Rutaceae | KAH9715793.1 |
| CmCCR | *Castanea mollissima* | Fagaceae | KAF3973871.1 |
| CpCCRL | *Ceratodon purpureus* | Ditrichaceae | [KAG0568142.1](https://www.ncbi.nlm.nih.gov/protein/KAG0568142.1?report=genbank&log$=protalign&blast_rank=1&RID=NVBXBX8A013) |
| CqCCR | *Chenopodium quinoa* | Amaranthaceae | XP_021717108.1 |
| CrCCR1 | *Ceratopteris richardii* | Pteridaceae | KAH7428812.1 |
| CrCCR2 | *Ceratopteris richardii* | Pteridaceae | [KAH7285939.1](https://www.ncbi.nlm.nih.gov/protein/KAH7285939.1?report=genbank&log$=protalign&blast_rank=1&RID=NVBXBX8A013) |
| DaCCR | *Dioscorea alata* | Dioscoreaceae | KAH7674983.1 |
| DcCCR1.1 | *Diphasiastrum complanatum* | Lycopodiaceae | [KAJ7550585.1](https://www.ncbi.nlm.nih.gov/protein/KAJ7550585.1?report=genbank&log$=protalign&blast_rank=1&RID=NVBXBX8A013) |
| DcCCR1.2 | *Diphasiastrum complanatum* | Lycopodiaceae | [KAJ7550386.1](https://www.ncbi.nlm.nih.gov/protein/KAJ7550386.1?report=genbank&log$=protalign&blast_rank=1&RID=NVBXBX8A013) |
| DcCCR2 | *Diphasiastrum complanatum* | Lycopodiaceae | [KAJ7514325.1](https://www.ncbi.nlm.nih.gov/protein/KAJ7514325.1?report=genbank&log$=protalign&blast_rank=1&RID=NVBXBX8A013) |
| EcCCR1 | *Eleusine coracana* | Poaceae | KAK3121815.1 |
| EcCCR2 | *Eleusine coracana* | Poaceae | KAK3123532.1 |
| EcCCR3 | *Eleusine coracana* | Poaceae | KAK3131788.1 |
| EgCCR | *Eucalyptus gunnii* | Myrtaceae | CAA56103.1 |
| HvCCR | *Hordeum vulgare* | Poaceae | XP_044948571.1 |
| LasCCR | *Lactuca sativa* | Asteraceae | KAJ0210860.1 |
| LleCCR | *Leucaena leucocephala* | Fabaceae | ABL01801.3 |
| MdCCR1.1 | *Malus domestica* | Rosaceae | XP_008361199.1 |
| MdCCR1.2 | *Malus domestica* | Rosaceae | XP_008375542.1 |
| MpCCRL | *Marchantia polymorpha* | Marchantiaceae | OAE30736.1 |
| MtCCR1 | *Medicago truncatula* | Fabaceae | NP_001411949.1 |
| MtCCR2 | *Medicago truncatula* | Fabaceae | NP_001411827.1 |
| OsCCR17 | *Oryza sativa* subsp. japonica | Poaceae | XP_015612613.1 |
| OsCCR19 | *Oryza sativa* subsp. japonica | Poaceae | XP_015651251.1 |
| OsCCR20 | *Oryza sativa* subsp. japonica | Poacea | XP_015650109.1 |
| OsCCR21 | *Oryza sativa* subsp. japonica | Poaceae | XP_015625865.1 |
| PhCCR | *Petunia hybrida* | Solanaceae | A0A059TC02.1 |
| PpCCR1.1 | *Prunus persica* | Rosaceae | XP_020415177.1 |
| PpCCR1.2 | *Prunus persica* | Rosaceae | XP_007215651.1 |
| PtCCR1 | *Populus tomentosa* | Salicaceae | AKG06582.1 |
| PtCCR7 | *Populus tomentosa* | Salicaceae | AGU43753.1 |
| PvaCCR1 | *Paspalum vaginatum* | Poaceae | KAJ1258320.1 |
| PvaCCR2 | *Paspalum vaginatum* | Poaceae | KAJ1268633.1 |
| PvCCR1 | *Panicum virgatum* | Poaceae | XP 039814571.1 |
| PvCCR2 | *Panicum virgatum* | Poaceae | ACZ74585.1 |
| QrCCR | *Quercus rubra* | Fagaceae | KAK4590115.1 |
| RcCCR | *Ricinus communis* | Euphorbiaceae | XP_002527302.1 |
| SbCCR1 | *Sorghum bicolor* | Poaceae | XP_002445566.1 |
| SbCCR2 | *Sorghum bicolor* | Poaceae | XP_002451664.1 |
| SfCCRL | *Sphagnum fallax* | Sphagnaceae | KAH8935327.1 |
| SlCCR1 | *Solanum lycopersicum* | Solanaceae | NP_001234612.1 |
| SlCCR2 | *Solanum lycopersicum* | Solanaceae | NP_001234297.1 |
| SmaCCRL | *Sphagnum magellanicum* | Sphagnaceae | KAH9534836.1 |
| SmoCCR1 | *Selaginella moellendorffii* | Selaginellaceae | ARC76937.1 |
| SmoCCR2 | *Selaginella moellendorffii* | Selaginellaceae | EFJ06720.1 |
| TaCCR1 | *Triticum aestivum* | Poaceae | ABE01883.1 |
| TaCCR2 | *Triticum aestivum* | Poaceae | XP_037433899.1 |
| TcCCR | *Theobroma cacao* | Malvaceae | EOY26038.1 |
| VfCCR1 | *Vicia faba* | Fabaceae | CAI8601404.1 |
| VfCCR2 | *Vicia faba* | Fabaceae | CAI8611802.1 |
| VvCCR | *Vitis vinifera* | Vitaceae | XP_002273454.1 |
| ZmCCR1 | *Zea mays* | Poaceae | XP_020408161.1 |
| ZmCCR2 | *Zea mays* | Poaceae | ACF85456.1 |
| ZmCCR3 | *Zea mays* | Poaceae | NP_001168852.1 |

**Table S2.** Accession numbers of double bond reductases (DBRs) in the phylogenetic tree.

| **Protein** | **Organism** | **Accession** |
| --- | --- | --- |
| AaDRB1 | *Artemisia annua* | ACN65116.1 |
| AtDBR | *Arabidopsis thaliana* | NP_197199.1 |
| AtceQORH | *Arabidopsis thaliana* | NP_193037.1 |
| AtENR | *Arabidopsis thaliana* | Q9SLA8 |
| CaDBR | *Colchicum autumnale* | UZM07734.1 |
| CsDBR2 | *Cannabis sativa* | KAF4379076.1 |
| CsDBR3 | *Cannabis sativa* | KAF4352490.1 |
| GsAER | *Gloriosa superba* | QLI49062.1 |
| MdENRL3 | *Malus domestica* | NP_001280847.1 |
| MdENRL5 | *Malus domestica* | NP_001281005.1 |
| MdHCDBR | *Malus domestica* | XP_028950755.1 |
| MdNCR1a | *Malus domestica* | XII92760.1 |
| MdNCR1b | *Malus domestica* | XII92761.1 |
| MpaDBR | *Marchantia paleacea* | AXN56964.1 |
| MpPulR | *Mentha × piperita* | Q6WAU0.1 |
| NtDBR | *Nicotiana tabacum* | 4HFJ_A |
| PaDBR1 | *Plagiochasma appendiculatum* | AGS43878.1 |
| PaDBR2 | *Plagiochasma appendiculatum* | AGS43879.1 |
| PtPPDBR | *Pinus taeda* | ABG91753.1 |
| RiRZS1 | *Rubus idaeus* | 6EOW_A |
| RiRZS2 | *Rubus idaeus* | AEL78826.1 |

**Table S3.** Accession numbers of aldehyde dehydrogenases (ALDHs) in the phylogenetic tree.

| **Protein** | **Organism** | **Accession** |
| --- | --- | --- |
| AaALDH1 | *Artemisia annua* | C5I9X1 |
| AtALDH3H1 | *Arabidopsis thaliana* | Q70DU8 |
| AtALDH12A1 | *Arabidopsis thaliana* | Q8VZC3 |
| AtALDH3I1 | *Arabidopsis thaliana* | Q8W033 |
| AtALDH10A8 | *Arabidopsis thaliana* | Q9S795 |
| AtALDH5F1 | *Arabidopsis thaliana* | Q9SAK4 |
| AtALDH10A9 | *Arabidopsis thaliana* | Q9STS1 |
| AtALDH6B2 | *Arabidopsis thaliana* | Q0WM29 |
| AtALDH11A3 | *Arabidopsis thaliana* | Q1WIQ6 |
| AtALDH2C4 | *Arabidopsis thaliana* | Q56YU0 |
| AtALDH2B7 | *Arabidopsis thaliana* | Q8S528 |
| AtALDH2B4 | *Arabidopsis thaliana* | Q9SU63 |
| AtALDH22A1 | *Arabidopsis thaliana* | Q0WSF1 |
| AtALDH3F1 | *Arabidopsis thaliana* | Q70E96 |
| AtALDH7B4 | *Arabidopsis thaliana* | Q9SYG7 |
| AmBALDH | *Antirrhinum majus* | C7A2A0 |
| CsALDH2B1 | *Camellia sinensis* | XP_028079149.1 |
| CsALDH2B2 | *Camellia sinensis* | XP_028121206.1 |
| CsALDH2B3 | *Camellia sinensis* | GMP82820.1 |
| CsALDH2B4 | *Camellia sinensis* | CAL5349916.1 |
| CsALDH2B5 | *Camellia sinensis* | GMQ10251.1 |
| CsALDH2C1 | *Camellia sinensis* | CAL5360597.1 |
| CsALDH2C2 | *Camellia sinensis* | GMQ06604.1 |
| CsALDH3F2 | *Camellia sinensis* | CAL5437890.1 |
| CsALDH3F3 | *Camellia sinensis* | XP_028062135.1 |
| CsALDH3H1 | *Camellia sinensis* | CAL5333237.1 |
| CsALDH3H2 | *Camellia sinensis* | CAL5348420.1 |
| CsALDH3I1 | *Camellia sinensis* | CAL5348420.1 |
| CsALDH5F1 | *Camellia sinensis* | XP_028124014.1 |
| CsALDH6B1 | *Camellia sinensis* | XP_028057398.1 |
| CsALDH6B2 | *Camellia sinensis* | GMP38830.1 |
| CsALDH7B1 | *Camellia sinensis* | XP_028108387.1 |
| CsALDH10A1 | *Camellia sinensis* | XP_028090466.1 |
| CsALDH11A | *Camellia sinensis* | XP_028090774.1 |
| CsALDH12A1 | *Camellia sinensis* | GMQ10500.1 |
| CsALDH18B1 | *Camellia sinensis* | XP_028065363.1 |
| CsALDH18B2 | *Camellia sinensis* | XP_028097620.1 |
| CsALDH22A1 | *Camellia sinensis* | XP_028060874.1 |
| GmALDH2B7 | *Glycine max* | XP_028194911.1 |
| GmALDH2B2 | *Glycine max* | XP_003528905.1 |
| GmALDH2B4 | *Glycine max* | KAH1246253.1 |
| GmALDH2C1 | *Glycine max* | KAH1234252.1 |
| GmALDH2D1 | *Glycine max* | KAG5041682.1 |
| GmALDH7A1 | *Glycine max* | AAP02957.1 |
| GmALDH7B1 | *Glycine max* | NP_001236813.2 |
| GmALDH7B8 | *Glycine max* | XP_003546512.1 |
| GmALDH10A4 | *Glycine max* | NP_001234990.1 |
| GmALDH10A5 | *Glycine max* | NP_001238427.1 |
| GmALDH11A3 | *Glycine max* | XP_003549550.1 |
| MdALDH7A1 | *Malus domestica* | Q9ZPB7 |
| MdAMADH1 | *Malus domestica* | A0A0E3T552 |
| MdAMADH2 | *Malus domestica* | A0A0E3T3B5 |
| OsBADH1 | *Oryza sativa subsp. japonica* | O24174 |
| OsBADH2 | *Oryza sativa subsp. japonica* | Q84LK3 |
| OsALDH5F1 | *Oryza sativa subsp. japonica* | B9F3B6 |
| PsAMADH2 | *Pisum sativum* | Q93YB2 |
| ScALDH2B2 | *Syntrichia caninervis* | AWK59819.1 |
| ScALDH10A1 | *Syntrichia caninervis* | AWK59820.1 |
| ScALDH11A1 | *Syntrichia caninervis* | AWK59821.1 |
| ScALDH21A1 | *Syntrichia caninervis* | ACT10823.1 |
| SlALDH2B1 | [*Solanum lycopersicum*](https://diurnal.sbs.ntu.edu.sg/species/view/1) | XP_010316283.1 |
| SlALDH2B7d | *Solanum lycopersicum* | NP_001333533.1 |
| SlALDH2B4 | *Solanum lycopersicum* | XP_004245256.1 |
| SlALDH3F1 | *Solanum lycopersicum* | NP_001333537.1 |
| SlALDH6B2 | *Solanum lycopersicum* | NP_001333575.1 |
| SlALDH3F1d | *Solanum lycopersicum* | XP_010316396.1 |
| SlALDH7B4 | *Solanum lycopersicum* | XP_004235550.2 |
| SlALDH2B7a | *Solanum lycopersicum* | NP_001333536.1 |
| SlALDH10A9 | *Solanum lycopersicum* | NP_001333606.1 |
| SlALDH12A1 | *Solanum lycopersicum* | NP_001333614.1 |
| SlALDH3H2 | *Solanum lycopersicum* | NP_001333570.1 |
| SlALDH11A3 | *Solanum lycopersicum* | XP_015082291.1 |
| SlALDH2C4 | *Solanum lycopersicum* | XP_004251601.1 |
| SlALDH5F1 | *Solanum lycopersicum* | NP_001233841.1 |
| SlALDH10A8 | *Solanum lycopersicum* | NP_001234235.2 |
| SoBADH | *Spinacia oleracea* | P17202 |
| TcALDH1 | *Tanacetum cinerariifolium* | A0A2I7G3B0 |
| VvALDH2B4 | *Vitis vinifera* | XP_002283132.1 |
| VvALDH2B9 | *Vitis vinifera* | XP_002274863.1 |
| VvALDH3F1 | *Vitis vinifera* | XP_002273358.2 |
| VvALDH3H1 | *Vitis vinifera* | CBI19000.3 |
| VvALDH3H5 | *Vitis vinifera* | XP_002273730.2 |
| VvALDH3J1 | *Vitis vinifera* | XP_002285466.1 |
| VvALDH5F1 | *Vitis vinifera* | XP_002265514.1 |
| VvALDH5F2 | *Vitis vinifera* | XP_019073007.1 |
| VvALDH5F3 | *Vitis vinifera* | XP_002265354.2 |
| VvALDH6B3 | *Vitis vinifera* | XP_002266390.1 |
| VvALDH6B5 | *Vitis vinifera* | XP_019074616.1 |
| VvALDH6B7 | *Vitis vinifera* | XP_010653335.1 |
| VvALDH7A1 | *Vitis vinifera* | XP_010656112.1 |
| VvALDH7D1 | *Vitis vinifera* | XP_010654421.1 |
| VvALDH10A9 | *Vitis vinifera* | XP_002283690.1 |
| VvALDH10A8 | *Vitis vinifera* | XP_002281984.1 |
| VvALDH11A3 | *Vitis vinifera* | XP_002285286.1 |
| VvALDH11B1 | *Vitis vinifera* | XP_002279374.1 |
| VvALDH12A1 | *Vitis vinifera* | XP_002273569.1 |
| VvALDH18B1 | *Vitis vinifera* | XP_010658318.1 |
| VvALDH18B3 | *Vitis vinifera* | XP_010654240.1 |
| VvALDH22A1 | *Vitis vinifera* | XP_002277743.1 |
| ZmAMADH1A | *Zea mays* | C0P9J6 |
| ZmAMADH2 | *Zea mays* | C6KEM4 |
| ZmAMADH1B | *Zea mays* | G5DDC2 |

**Table S4.** Accession numbers of 4-coumaroyl:CoA ligases (4CLs) in the phylogenetic tree.

| **Protein** | **Organism** | **Accession** |
| --- | --- | --- |
| At4CL1 | *Arabidopsis thaliana* | AAA82888.1 |
| At4CL2 | *Arabidopsis thaliana* | AAD47193.1 |
| At4CL3 | *Arabidopsis thaliana* | AAD47195.1 |
| At4CL4 | *Arabidopsis thaliana* | AXN70030.1 |
| Cs4CL1 | *Camellia sinensis* | CAL5440474.1 |
| Cs4CL2 | *Camellia sinensis* | XP_028078731.1 |
| Cs4CL3 | *Camellia sinensis* | GMP70894.1 |
| Gm4CL1 | *Glycine max* | AAL98709.1 |
| Gm4CL2 | *Glycine max* | AAC97600.1 |
| Gm4CL3 | *Glycine max* | AAC97599.1 |
| Gm4CL4 | *Glycine max* | CAC36095.1 |
| Ler4CL1 | *Lithospermum erythrorhizon* | BAA08365.1 |
| Ler4CL2 | *Lithospermum erythrorhizon* | BAA08366.2 |
| Lle4CL1 | *Leucaena leucocephala* | ACI23348.1 |
| Lle4CL2 | *Leucaena leucocephala* | ACI23349.1 |
| Mn4CL1 | *Morus notabilis* | EXB80937.1 |
| Mn4CL2 | *Morus notabilis* | XP_010092099.1 |
| Mn4CL3 | *Morus notabilis* | XP_010087481.1 |
| Mp4CL1 | *Marchantia paleacea* | WWQ35057.1 |
| Mp4CL2 | *Marchantia paleacea* | WWQ35058.1 |
| Mp4CL3 | *Marchantia paleacea* | WWQ35059.1 |
| Mp4CL4 | *Marchantia paleacea* | WWQ35060.1 |
| Nt4CL1 | *Nicotiana tabacum* | AAB18637.1 |
| Nt4CL2 | *Nicotiana tabacum* | AAB18638.1 |
| Os4CL1 | *Oryza sativa* | NP_001061353.1 |
| Os4CL2 | *Oryza sativa* | NP_001047819.1 |
| Os4CL3 | *Oryza sativa* | NP_001046069.1 |
| Os4CL4 | *Oryza sativa* | NP_001058252.1 |
| Os4CL5 | *Oryza sativa* | NP_001061935.1 |
| Pa4CL | *Plagiochasma appendiculatum* | AJT43268.1 |
| Pl4CL1 | *Pueraria lobata* | AGW16013.1 |
| Pl4CL2 | *Pueraria lobate* | AGW16014.1 |
| Pp4CL1 | *Physcomitrium patens* | ABV60447 |
| Pp4CL2 | *Physcomitrium patens* | ABV60448 |
| Pp4CL3 | *Physcomitrium patens* | ABV60449 |
| Pp4CL4 | *Physcomitrium patens* | ABV60450 |
| Pto4CL1 | *Populus tomentosa* | AAL02145.1 |
| Pto4CL2 | *Populus tomentosa* | AFC89538.1 |
| Pto4CL3 | *Populus tomentosa* | AFC89539.1 |
| Pto4CL4 | *Populus tomentosa* | AFC89540.1 |
| Pto4CL5 | *Populus tomentosa* | AFC89541.1 |
| Ptre4CL1 | *Populus tremuloides* | AAC24503.1 |
| Ptre4CL2 | *Populus tremuloides* | AAC24504.1 |
| Ptri4CL1 | *Populus trichocarpa* | XP_002304825.2 |
| Ptri4CL2 | *Populus trichocarpa* | ACC63868.1 |
| Ptri4CL3 | *Populus trichocarpa* | XP_002297699.2 |
| Ptri4CL4 | *Populus trichocarpa* | XP_002325815.1 |
| Ptri4CL5 | *Populus trichocarpa* | ACC63867.1 |
| Pv4CL1 | *Panicum virgatum* | ACD02135.1 |
| Pv4CL2 | *Panicum virgatum* | ADZ96250.1 |
| Rg4CL1 | *Rehmannia glutinosa* | QYK93270.1 |
| Rg4CL2 | *Rehmannia glutinosa* | QYK93271.1 |
| Ri4CL1 | *Rubus idaeus* | AAF91310.1 |
| Ri4CL2 | *Rubus idaeus* | AAF91309.1 |
| Ri4CL3 | *Rubus idaeus* | AAF91308.1 |
| Sa4CL1 | *Sorbus aucuparia* | ADF30254.1 |
| Sa4CL2 | *Sorbus aucuparia* | ADE96996.1 |
| Sa4CL3 | *Sorbus aucuparia* | ADE96997.1 |
| Sb4CL1 | *Sorghum bicolor* | XP_002444107 |
| Sb4CL2 | *Sorghum bicolor* | XP_002452704 |
| Sb4CL3 | *Sorghum bicolor* | XP_002451647 |
| Sb4CL4 | *Sorghum bicolor* | XP_002438783 |
| Sma4CL1 | *Salvia miltiorrhiza* | AAP68990.1 |
| Sma4CL2 | *Salvia miltiorrhiza* | AAP68991.1 |
| Smo4CL1 | *Selaginella moellendorffii* | EFJ13708.1 |

**Table S5.** Accession numbers of glycosyltransferases (GTs) in the phylogenetic tree.

| **Protein** | **Organism** | **Accession** |
| --- | --- | --- |
| AcA3GT | *Aralia cordata* | BAD06514.1 |
| Am4C’GT | *Antirrhinum majus* | Q33DV3 |
| AmF7GT | *Antirrhinum majus* | BAG16514.1 |
| AtF3GT | *Arabidopsis thaliana* | NP_564357.1 |
| BvF7GT | *Beta vulgaris* | AAS94330.1 |
| CcM3GT | *Crocosmia × crocosmiiflora* | A0A2Z5CVA1.1 |
| CpF3GT | *Citrus × paradisi* | ACS15351.1 |
| CsF37GT | *Camellia sinensis* | ALO19886.1 |
| CsF3GT1 | *Camellia sinensis* | CAL5376570.1 |
| CsF3GT2 | *Camellia sinensis* | ALO19888.1 |
| CtA3GT1 | *Clitoria ternatea* | BAF49298.1 |
| CtA3GT2 | *Clitoria ternatea* | 3WC4_A |
| DcF3GT | *Dianthus caryophyllus* | BAD52003.1 |
| FaF3GT1 | *Fragaria × ananassa* | Q2V6K0.1 |
| FaF3GT2 | *Fragaria × ananassa* | Q2V6J9.1 |
| GhA5GT | *Glandularia × hybrida* | Q9ZR25.1 |
| GmF3GT | *Glycine max* | XP_003533968.1 |
| GmIF7GT | *Glycine max* | A6BM07 |
| GtF3GT | *Gentiana triflora* | Q96493.1 |
| GuF3GT | *Glycyrrhiza uralensis* | QDM38894.1 |
| IhA3GT | *Iris x hollandica* | BAD83701.1 |
| MdF3GT1 | *Malus domestica* | XP_008362332.2 |
| MdF3GT2 | *Malus domestica* | XP_008380456.1 |
| MdP2’GT | *Malus domestica* | B3TKC8.1 |
| MdP4’GT | *Malus domestica* | QLF96270.1 |
| MrF3GT | *Morella rubra* | KAB1224445.1 |
| MtF3GT | *Medicago truncatula* | XP_003610163.1 |
| NtF7GT | *Nicotiana tabacum* | BAB88935.1 |
| PcF7GT | *Pyrus communis* | AAY27090.1 |
| PcP2′GT | *Pyrus communis* | D3UAG3.1 |
| PfA5GT | *Perilla frutescens* | Q9ZR27.1 |
| PfF3GT | *Perilla frutescens* | BAA19659.1 |
| PhA5GT | *Petunia × hybrida* | BAA89009.1 |
| PhK3GT | *Petunia × hybrida* | Q9SBQ8.1 |
| PpF3GT1 | *Prunus persica* | XP_007217952.1 |
| PpF3GT2 | *Prunus persica* | XP_007224129.2 |
| RhA5,3GT | *Rosa hybrid cultivar* | Q4R1I9.1 |
| SbF3GT | *Scutellaria baicalensis* | QBL54224.1 |
| SgF3,7GT1 | *Siraitia grosvenorii* | AXK92493.1 |
| SgF3,7GT2 | *Siraitia grosvenorii* | AXK92492.1 |
| UGT71A15 | *Malus domestica* | NP_001315903.1 |
| UGT71K1 | *Malus domestica* | NP_001280899.1 |
| UGT75L17 | *Malus domestica* | NP_001315912.1 |
| UGT88F4 | *Malus domestica* | ARV88477.1 |
| VaA5GT | *Vitis amurensis* | AHL68667.1 |
| VvA3GT | *Vitis vinifera* | P51094.2 |

**Table S6.** Primer pairs used for gene cloning*.

| **Gene** | **Primer** | **Sequence (5'🡪3')** |
| --- | --- | --- |
| *LlCCR* | Forward | ggtaggcatatggagATGCCAGTTTCTGGACAAAC |
|  | Reverse | ctcgagggtaccgagTTAAGACTGAATATGAATAGAATCTTC |
| *LlCCR-Like1* | Forward | ggtaggcatatggagATGAGCGGTGGAGCT |
|  | Reverse | ctcgagggtaccgagTTAAAAACTGACAAACTTCTTTTCC |
| *LlCCR-Like2* | Forward | ggtaggcatatggagATGAGTGGAGCAGAGTC |
|  | Reverse | ctcgagggtaccgagTTAGACACTTAAAAAGTTCTTCTC |
| *LlCCR-Like3* | Forward | ggtaggcatatggagATGGCAGCTGAGAACGAG |
|  | Reverse | ctcgagggtaccgagTCAATCAACGAGTCCAGC |
| *LlCCR-Like4* | Forward | ggtaggcatatggagATGGCAAAAGACGGAGAG |
|  | Reverse | ctcgagggtaccgagTCAAGAAATAAATCCTTTGCTC |
| *LlCCR-Like5* | Forward | ggtaggcatatggagATGGTAATGGTAGTGGAAGAAG |
|  | Reverse | ctcgagggtaccgagTCACTGATTGCATGAAAAACTTTG |
| *LlCCR-Like6* | Forward | ggtaggcatatggagATGGCACCAGCTTCTTTCC |
|  | Reverse | ctcgagggtaccgagTCACGATGAGTTTTCATCAGTCC |
| *LlCCR-Like7* | Forward | ggtaggcatatggagATGGGGATTGTAAGGACGG |
|  | Reverse | ctcgagggtaccgagTTAACATTCACTGTAACAACTCC |
| *LlCCR-Like8* | Forward | ggtaggcatatggagATGAGTGGAGAGGAGAAGG |
|  | Reverse | ctcgagggtaccgagTCAAATAGTGATGATGCCG |
| *LlCCR-Like9* | Forward | ggtaggcatatggagATGAAAGAACAAGAAGCAAAAAG |
|  | Reverse | ctcgagggtaccgagCTAACATGCAGTGCCCTC |
| *LlCCR-Like10* | Forward | ggtaggcatatggagATGACAGAGGTAGAAGGG |
|  | Reverse | ctcgagggtaccgagCTAGTCCTTACTCAGGAAAC |
| *LlCCR-Like11* | Forward | ggtaggcatatggagATGGCCGCTGAGAGC |
|  | Reverse | ctcgagggtaccgagTCAATCAATGAGTCCAGCCTT |
| *LlCHS1* | Forward | ggtaggcatatggagATGGTGACTGTTGATGAAG |
|  | Reverse | ctcgagggtaccgagTTAAGCAGAGACACTGTGG |
| *LlDBR1* | Forward | ggtaggcatatggagATGGCAAGTGGTGGTGG |
|  | Reverse | ctcgagggtaccgagTCATTCTTGAGCAACTAAAACTACC |
| *LlDBR2* | Forward | ggtaggcatatggagATGGCAGGTGTGGTTGAG |
|  | Reverse | ctcgagggtaccgagTTATTTCTTAACTTCAATTATAAC |
| *LlDBR3* | Forward | ggtaggcatatggagATGGCAAGTGGTGGTGGTG |
|  | Reverse | ctcgagggtaccgagTCATTCTTGAGCAACTAAAAC |
| *LlDBR4* | Forward | ggtaggcatatggagATGGCAAGTGGTGGTGGTG |
|  | Reverse | ctcgagggtaccgagTCATTCTTGAGCAACTA |
| *LlDBR5* | Forward | ggtaggcatatggagATGGAGGAAGTGGACAACAAG |
|  | Reverse | ctcgagggtaccgagTCATTCAGAGGCTACTCGAATGAC |
| *LlDBR6* | Forward | ggtaggcatatggagATGGAAGTGACCAACAGGTAC |
|  | Reverse | ctcgagggtaccgagTCACTCATCTGCAAGCTTAAC |
| *LlALDH1* | Forward | ggtaggcatatggagATGAACATCTCTATGGAGCTTTTG |
|  | Reverse | ctcgagggtaccgagTCACCATCCAATCAAAGCAAG |
| *LlALDH2* | Forward | ggtaggcatatggagATGGGGTCTGTGGAGAAAG |
|  | Reverse | ctcgagggtaccgagTTAATTCTTTGACAATCCGATCAG |
| *LlALDH3* | Forward | ggtaggcatatggagATGGGGAGTCATGGTAATG |
|  | Reverse | ctcgagggtaccgagTCAAAGCCAAGGAGAATTATATAG |
| *LlALDH4* | Forward | ggtaggcatatggagATGAGTACGGAGGTTGAG |
|  | Reverse | ctcgagggtaccgagTTAATTGCTGTTCATATTCCCC |
| *LlALDH5* | Forward | ggtaggcatatggagATGGCCATCCAAATCCCTTC |
|  | Reverse | ctcgagggtaccgagCTAGTTTTGCTTTTGGCTTCTTTC |
| *LlALDH6* | Forward | ggtaggcatatggagATGGAGACTTTGAGAGACTTCG |
|  | Reverse | ctcgagggtaccgagCTATATGTCAATTAATCCATGCCTA |
| *Ll4CL1* | Forward | ggtaggcatatggagATGGCTCTCCAAACAAAGGAA |
|  | Reverse | ctcgagggtaccgagTCACTTTGAAAAGTCGGCTG |
| *Ll4CL2* | Forward | ggtaggcatatggagATGATTTCTGTTGCTTCATC |
|  | Reverse | ctcgagggtaccgagCTAAGGCATAGGGGAGGCTG |
| *Ll4CL3* | Forward | ggtaggcatatggagATGGAGTCCCAAAAAGACCTC |
|  | Reverse | ctcgagggtaccgagTTAATTTGCTAGACCTGCTGC |
| *Ll4CL4* | Forward | ggtaggcatatggagATGGACCCAAACAGCGGTTTC |
|  | Reverse | ctcgagggtaccgagTCATAACCTAGAAGAAGAGTTG |
| *LlP4′GT* | Forward | ggtaggcatatggagATGGAAGCGATAGTTCTGTAC |
|  | Reverse | ctcgagggtaccgagTCACTTCCACGACTCAAC |
| *LlP2′GT1* | Forward | ggtaggcatatggagATGGAGACCATAGTATTGTATC |
|  | Reverse | ctcgagggtaccgagTCATCCTCGCTTCCATG |
| *LlP2′GT2* | Forward | ggtaggcatatggagATGCAAGACACGATAGTCC |
|  | Reverse | ctcgagggtaccgagCTACCCAAGTGCCTCAAC |
| *LlP2′GT3* | Forward | ggtaggcatatggagATGGAGGATGCCATAGTTC |
|  | Reverse | ctcgagggtaccgagTTAGTTTTGCCTCCACAACTC |

*****. *CCR*, *cinnamoyl CoA reductase*; *CHS*, *chalcone synthase*; *DBR*, *double bond reductase*; *ALDH*, *aldehyde dehydrogenase*; *4CL*, *4-coumaroyl: CoA ligase*; *P4′GT*, *phloretin 4′-O-glucosyltransferase*; *P2′GT*, *phloretin 2′-O-glucosyltransferase*. The lowercase letters in the primers indicated the homologous sequences of the end of the vector designed for homologous recombination.

**Table S7.** Oligonucleotides used for gene expression suppression*.

| **Gene** | **Usage** | **Sequence (5'🡪3')** |
| --- | --- | --- |
| *LlCCR* | asODN | atttctaccacctctccacg |
|  | sODN(control) | cgtggagaggtggtagaaat |
| *LlDBR1* | asODN | accctgtgttgttgtaatga |
|  | sODN(control) | tcattacaacaacacagggt |
| *LlALDH1* | asODN | gctccagtagtgcagatgtt |
|  | sODN(control) | aacatctgcactactggagc |
| *Ll4CL2* | asODN | gtgtttcttgggtttggggc |
|  | sODN(control) | gccccaaacccaagaaacac |
| *LlP4′GT* | asODN | gtggtgtgtgagtattagtt |
|  | sODN(control) | aactaatactcacacaccac |
| *LlP2′GT1* | asODN | gtgcagggaagggtggtaag |
|  | sODN(control) | ctttttaccacccttccctgtctatc |

**.* Underlined letters indicated thiophosphorylation-modified nucleotides for stability improvement. *CCR*, *cinnamoyl CoA reductase*; *DBR*, *double bond reductase*; *ALDH*, *aldehyde dehydrogenase*; *4CL*, *4-coumaroyl:CoA ligase*; *P4′GT*, *phloretin 4′-O-glucosyltransferase*; *P2′GT*, *phloretin 2′-O-glucosyltransferase*.

**Table S8.** Primers used for gene expression*.

| **Gene** | **Primer** | **Sequence (5'🡪3')** |
| --- | --- | --- |
| *β-actin* | Forward | TGGTATGGAAGCTCCGGGTA |
|  | Reverse | GGTTGACCCACCACTAAGCA |
| *LlPAL1* | Forward | ATTGGTGAAGCTCGGAGGTG |
|  | Reverse | CCATGCTCTCCAGAACCCAG |
| *LlPAL2* | Forward | ACACATGAGCTCAAGCACCA |
|  | Reverse | TGCTTTGGCTTTGTAAGTGGG |
| *LlC4H* | Forward | AGATTGCAGCTCTTCAAGGAGAA |
|  | Reverse | AAACTTTCATGGTCAGTGGCCT |
| *Ll4CL1* | Forward | TAGGTGCTACAACCACTGCG |
|  | Reverse | TGAGTTTTGCGTTGGACCCT |
| *Ll4CL2* | Forward | GCTCCATTAGGGTGGTGCTT |
|  | Reverse | GCCTAAAATCGCCTGAGGAA |
| *Ll4CL3* | Forward | ATCACGTAAAGTGGCCTCGG |
|  | Reverse | GGGTTCGCAGTCGTGAGTAT |
| *Ll4CL4* | Forward | GTCGAAGTGAGCCAGACCGA |
|  | Reverse | TCCGGTGCGCGTAGGTG |
| *LlHCT* | Forward | TTGGAACTTCGCCAGCTCAT |
|  | Reverse | GCATGCCAACACCAAGTGAG |
| *LlCAD* | Forward | CAGGGAGGCTTTGCTAGCTC |
|  | Reverse | TAGTGGTGCTGCCTGTTCTG |
| *LlCCR* | Forward | AGTTCTTCCCTGAGTATCCGA |
|  | Reverse | CGTGAACTCTAAGCCCAAGTC |
| *LlCHS1* | Forward | AGGCATTCCAACCTTTGGGT |
|  | Reverse | AGGCTTGAGGCCCAATTTGA |
| *LlCHS2* | Forward | CAGAAGGTCCAGCCACCATT |
|  | Reverse | TGTGCTCGCTCTTGGTGATT |
| *LlCHI1* | Forward | TGGCCTTTACACAGAGGCAG |
|  | Reverse | ATAGAAGAGCCGGGGGTGAA |
| *LlCHI2* | Forward | CCTGGCAATGTCGTAGCTGA |
|  | Reverse | TCGATCCCTCACCGAACTCT |
| *LlDRB1* | Forward | AGGCTCCAACGCTATTCTGG |
|  | Reverse | ATCCATCAATGGGCGAACCA |
| *LlDBR2* | Forward | TATGCACCCGAAGGTGTTCC |
|  | Reverse | GCCAGACATCAGGGTACGTT |
| *LlALDH1* | Forward | ACAAAGCCCCTTGCTGCATA |
|  | Reverse | TCATTGATGGCCAATCCCCC |
| *LlALDH2* | Forward | TCCTTTGTCCACCTTATCAGAACT |
|  | Reverse | CTGCTTTGTCTACATCAAGCCT |
| *LlP4′GT* | Forward | ACACCCTCCTTATCGTTCCTG |
|  | Reverse | GCCATGGTGATTGAGCCG |
| *LlP2′GT1* | Forward | CTCCTTGGAAGTTTTCCTGTTTG |
|  | Reverse | GGAGTGAAGTGGCGATTTTGAG |
| *LlP2′GT2* | Forward | CACAGGCACCTTCCAAAGCA |
|  | Reverse | TTCGAGTTGGGGTTGTGTCAA |

**. PAL*, *phenylalanine ammonia lyase*; *C4H*, *cinnamate 4-hydroxylase*; *4CL*, *4-coumaroyl:CoA ligase*; *HCT*, *hydroxycinnamoyl transferase*; *CAD*, *cinnamyl alcohol dehydrogenase*; *CCR*, *cinnamoyl CoA reductase*; *CHS*, *chalcone synthase*; *CHI*, *chalcone synthase*; *DBR*, *double bond reductase*; *ALDH*, *aldehyde dehydrogenase*; *P4′GT*, *phloretin 4′-O-glucosyltransferase*; *P2′GT*, *phloretin 2′-O-glucosyltransferase*.


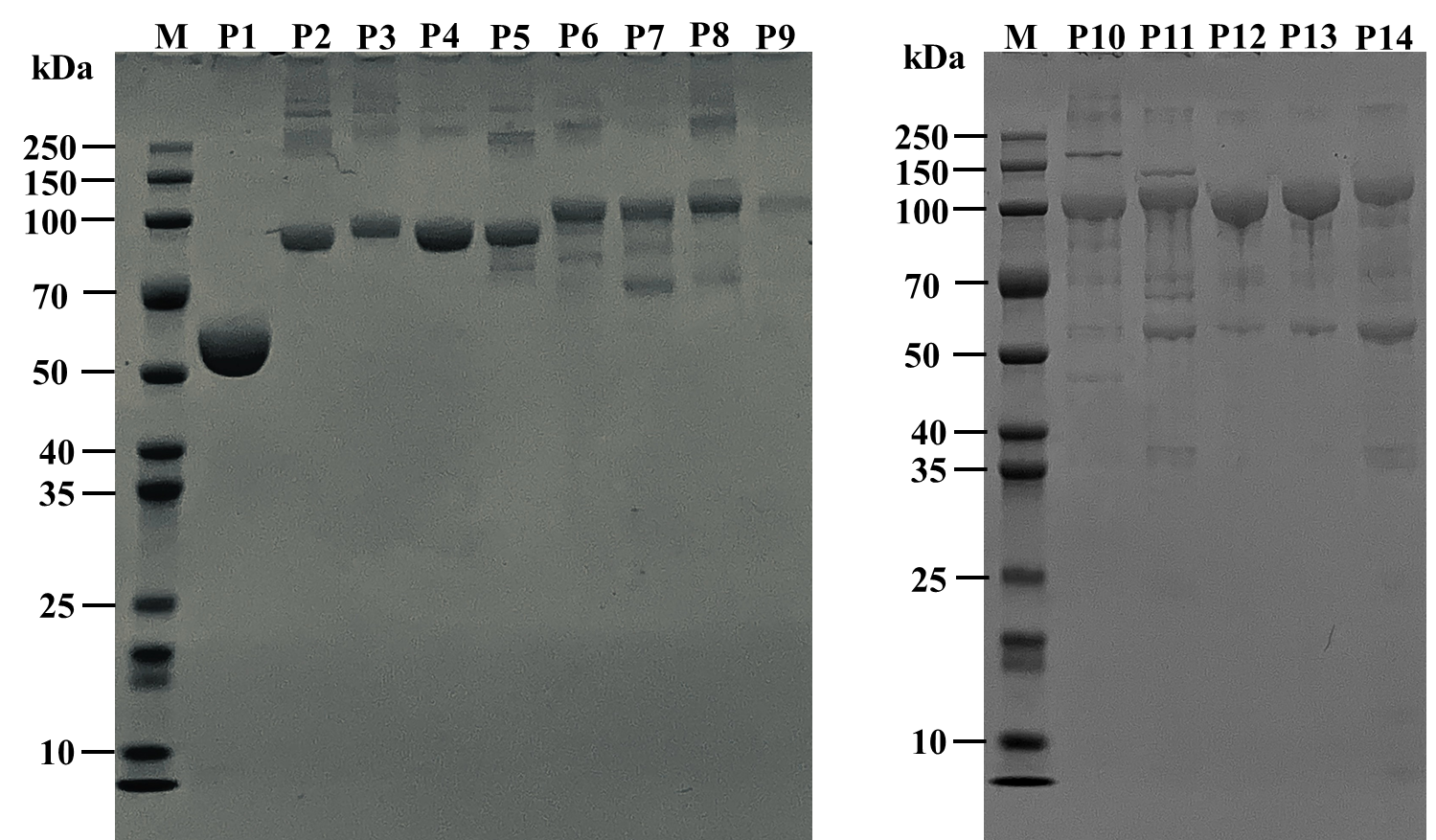


**Fig. S1.** SDS-PAGE analysis of the purified recombinant protein expressed in *E. coli*. M, protein marker; P1, empty protein TF (including his-tag, trigger factor and cutoff site of HRV 3C Protease) obtained from expression of the empty vector, molecular weight (MW)=54.39 kDa (after insertion of the exogenous gene with stop codon, the actual MW of TF was 52.52 kDa); P2, LlCCR, MW=88.93 kDa (36.41 kDa + TF); P3, LlCHS1, MW=95.26 kDa (42.74 kDa + TF); P4, LlDBR1, MW=91.31 kD (38.79 kDa + TF); P5, LlDBR2, MW=90.92 kDa (38.40 kDa + TF); P6, LlP4′GT1, MW=103.86 kDa (51.34 kDa+ TF); P7, LlP2′GT1, MW=104.74 kDa (52.22 kDa + TF); P8, LlP2′GT2 MW=106.35 kDa (53.83 kDa + TF); P9, LlP2′GT3, MW=105.19 kDa (52.67 kDa + TF); P10, LlALDH1 MW=105.87 kDa (53.35 kDa + TF); P11, LlALDH2 MW=112.48 kDa (59.96 kDa + TF); P12, LlALDH3 MW=106.89 kDa (54.37 kDa + TF); P13, Ll4CL1 MW=112.00 kDa (59.48 kDa + TF); P14, Ll4CL2 MW=114.98 kDa (62.46 kDa + TF).


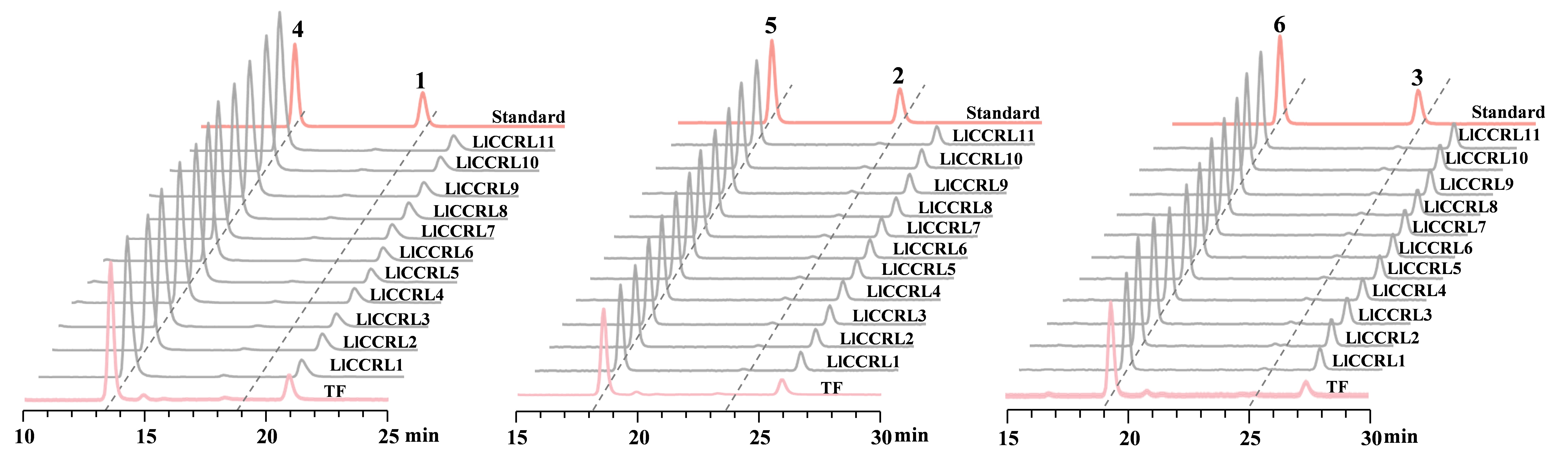


**Fig. S2.** Enzyme activity of cinnamoyl CoA reductase-like proteins (LlCCRL1~11) from *Lithocarpus litseifolius* towards different substrates. The expressed empty vector protein (TF) was used as control. The reactions were performed at 30°C and at pH 6.0 for 30 min, and terminated by addition of NaOH. NaOH released the CoA esters of the substrates as their acid forms in the reaction solution for HPLC detection. p-Coumaroyl-CoA (pCCoA), feruloyl-CoA (FCoA), and sinapoyl-CoA (ScoA) substrates were tested, and the product p-coumaraldehyde (pCAld), coniferyl aldehyde (CAld) and sinapoyl aldehyde (SAld) were recorded at 280 nm on a 20A Shimadzu HPLC. Peak 1, pCAld; peak 2, CAld; peak 3, SAld; peak 4, p-coumaric acid; peak 5, ferulic acid; peak 6, sinapic acid.


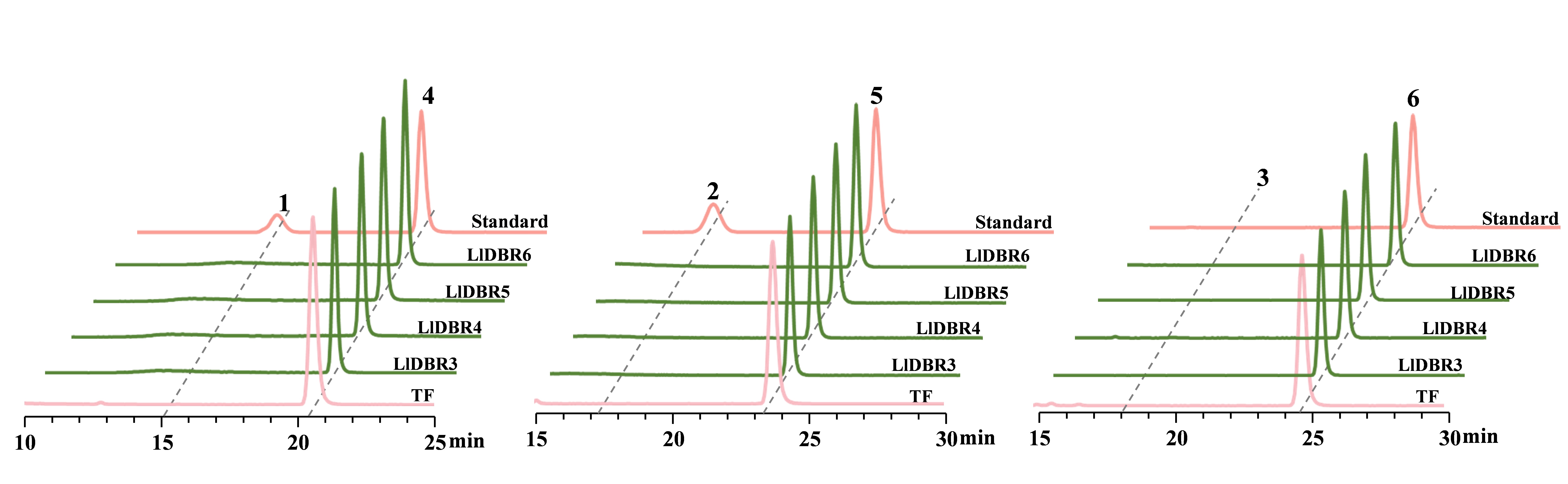


**Fig. S3**. Enzyme activity of double bond reductase (LlDBR3~6) from *Lithocarpus litseifolius* towards different substrates. The expressed empty vector protein (TF) was used as control. The reactions were performed at 30°C and pH 6.5 for 30 min and terminated by addition of trifluoroacetic acid. p-Coumaraldehyde (pCAld), coniferyl aldehyde (CAld) and sinapoyl aldehyde (SAld) substrates were tested, and the product dihydro-p-coumaraldehyde (dihydro-pCAld), dihydro-coniferyl aldehyde (dihydro-CAld) and dihydro-sinapoyl aldehyde (dihydro-SAld) were recorded at 280 nm after HPLC separation. Peak 1, dihydro-pCAld; peak 2, dihydro-CAld; peak 3, dihydro-SAld; peak 4, pCAld; peak 5, CAld; peak 6, SAld.


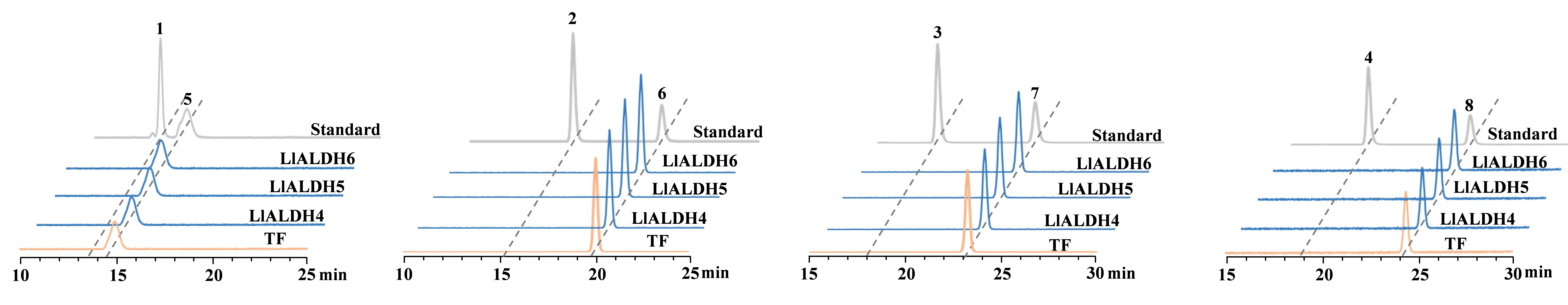


**Fig. S4**. Enzyme activity of aldehyde dehydrogenases (LlALDH4~6) from *Lithocarpus litseifolius* towards different substrates. The expressed empty vector protein (TF) was used as control. The reactions were performed at 30°C and at pH 6.5 for 30 min, and terminated by addition of trifluoroacetic acid. Dihydro-*p*-coumaraldehyde (dihydro-pCAld), *p*-coumaraldehyde (pCAld), coniferaldehyde (CAld) and sinapaldehyde (SAld) substrates were tested, and the product dihydro-*p*-coumaric acid (dihydro-pCAci), *p*-coumaric acid (pCAci), ferulic acid (FAci), and sinapic acid (SAci) were recorded at 280 nm on a 20AD Shimadzu HPLC. Peak 1, dihydro-pCAci; peak 2, pCAci; peak 3, FAci; peak 4, SAci; peak 5, dihydro-pCAld; peak 6, pCAld; peak 7, CAld; peak 8, SAld.

**
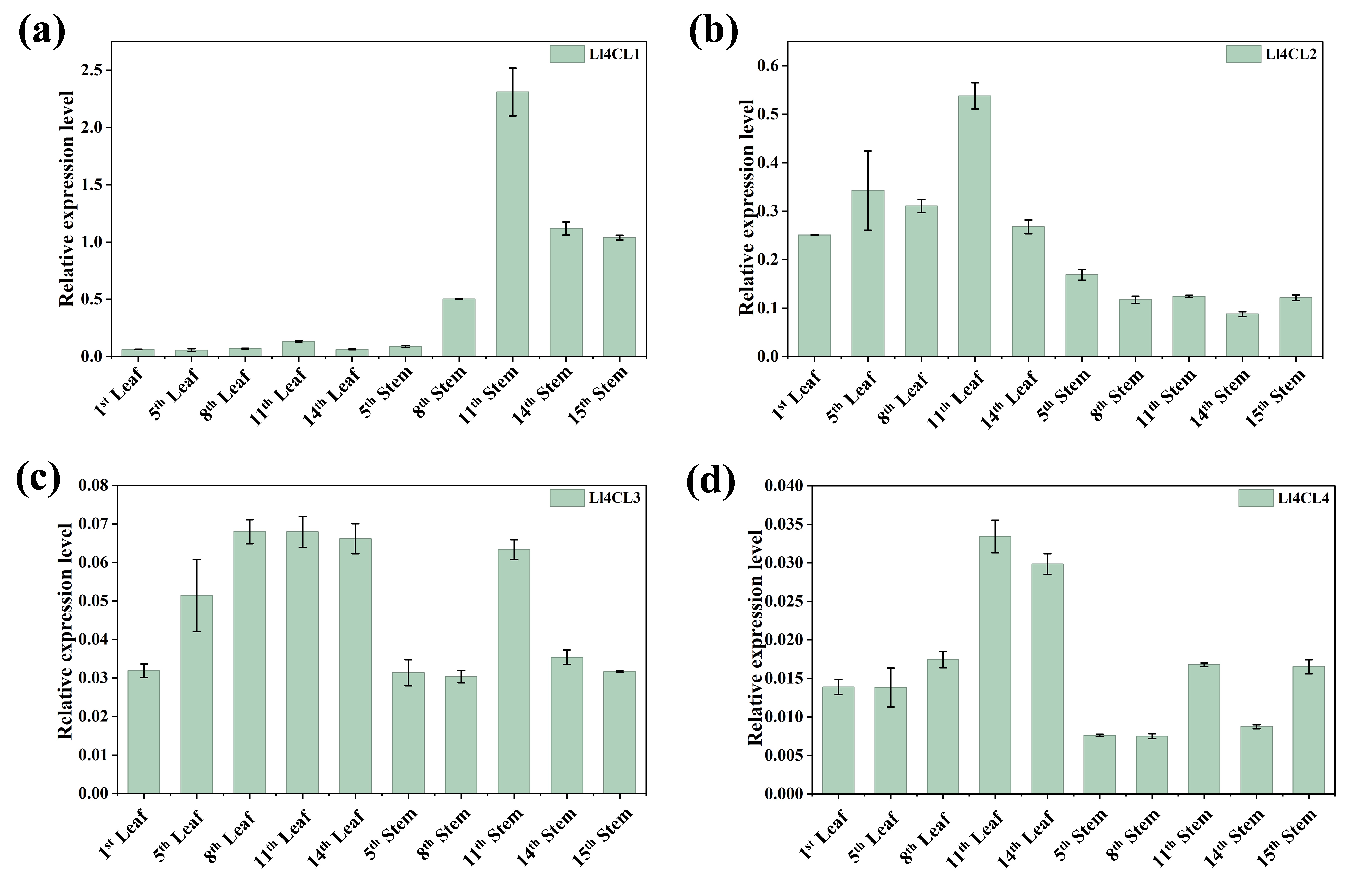
**

**Fig. S5.** Expression levels of the *4-coumaroyl:CoA ligases* (*4CLs*) in various leaves and stems on the newly born shoots of *Lithocarpus litseifolius.* (a) *Ll4CL1*, (b) *Ll4CL2*, (c) *Ll4CL3*, (d) *Ll4CL4*. Relative expression levels of the genes were calculated using 2^-△CT^, and presented as mean ± SD of three repetitions.


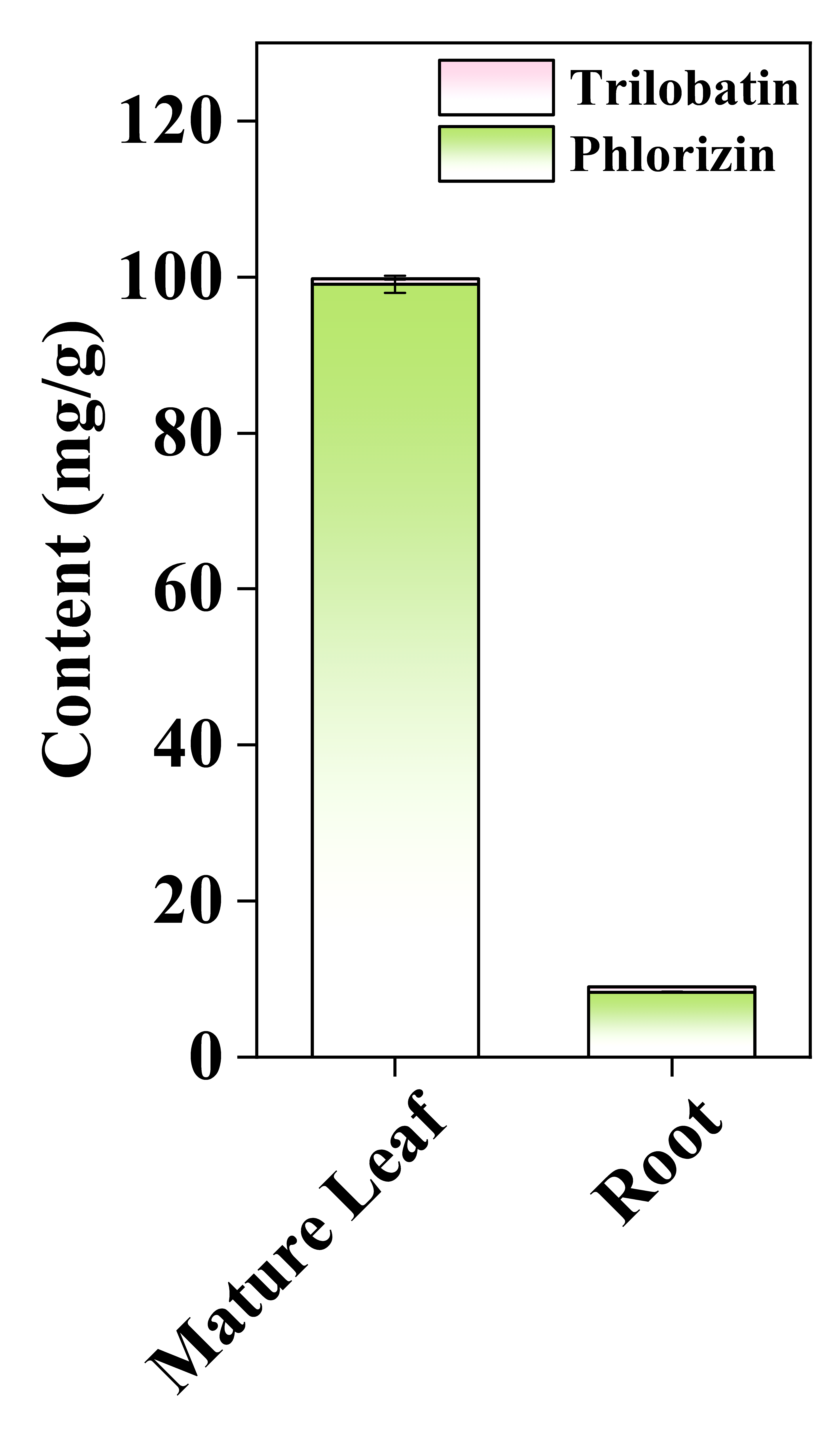


**Fig. S6.** DHC profiles in the mature leaf and root tip. Data are means ± SD of three replicates.
